# Pharmaceutical metabolite identification in lettuce (*Lactuca sativa*) and earthworms (*Eisenia fetida*) using liquid chromatography coupled to high-resolution mass spectrometry and in silico spectral library

**DOI:** 10.1007/s00216-024-05515-2

**Published:** 2024-09-10

**Authors:** Jan Fučík, Stanislav Fučík, Sascha Rexroth, Marian Sedlář, Helena Zlámalová Gargošová, Ludmila Mravcová

**Affiliations:** 1 Institute of Chemistry and Technology of Environmental Protection, Faculty of Chemistry, Brno University of Technology, Purkyňova 118, 612 00 Brno, Czech Republic; 2 Faculty of Electrical Engineering and Communication, Brno University of Technology, Technická 3058/10, 616 00 Brno, Czech Republic; 3 Shimadzu Europa GmbH, Albert-Hahn-Straße 6, 472 69 Duisburg, Germany; 4 CEITEC Brno University of Technology, Purkyňova 656/123, 612 00 Brno, Czech Republic

**Keywords:** Pharmaceuticals, Software prediction, Metabolite identification in *Eisenia fetida* and *Lactuca sativa*, Liquid chromatography, High-resolution mass spectrometry, In silico spectral library

## Abstract

Pharmaceuticals released into the aquatic and soil environments can be absorbed by plants and soil organisms, potentially leading to the formation of unknown metabolites that may negatively affect these organisms or contaminate the food chain. The aim of this study was to identify pharmaceutical metabolites through a triplet approach for metabolite structure prediction (software-based predictions, literature review, and known common metabolic pathways), followed by generating in silico mass spectral libraries and applying various mass spectrometry modes for untargeted LC-qTOF analysis. Therefore, *Eisenia fetida* and *Lactuca sativa* were exposed to a pharmaceutical mixture (atenolol, enrofloxacin, erythromycin, ketoprofen, sulfametoxazole, tetracycline) under hydroponic and soil conditions at environmentally relevant concentrations. Samples collected at different time points were extracted using QuEChERS and analyzed with LC-qTOF in data-dependent (DDA) and data-independent (DIA) acquisition modes, applying both positive and negative electrospray ionization. The triplet approach for metabolite structure prediction yielded a total of 3762 pharmaceutical metabolites, and an in silico mass spectral library was created based on these predicted metabolites. This approach resulted in the identification of 26 statistically significant metabolites (*p* < 0.05), with DDA + and DDA − outperforming DIA modes by successfully detecting 56/67 sample type:metabolite combinations. Lettuce roots had the highest metabolite count (26), followed by leaves (6) and earthworms (2). Despite the lower metabolite count, earthworms showed the highest peak intensities, closely followed by roots, with leaves displaying the lowest intensities. Common metabolic reactions observed included hydroxylation, decarboxylation, acetylation, and glucosidation, with ketoprofen-related metabolites being the most prevalent, totaling 12 distinct metabolites. In conclusion, we developed a high-throughput workflow combining open-source software with LC-HRMS for identifying unknown metabolites across various sample types.

**Graphical Abstract:**

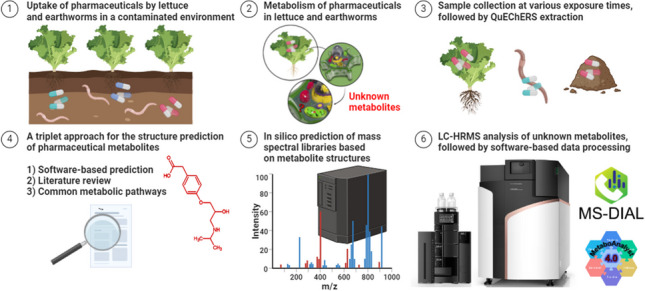

**Supplementary Information:**

The online version contains supplementary material available at 10.1007/s00216-024-05515-2.

## Introduction

Pharmaceuticals play a crucial role in human healthcare and animal husbandry; however, a significant challenge arises as up to 90% of the drug dosage can be excreted in feces or urine. Unfortunately, conventional wastewater treatment plants are not entirely effective in removing these organic micropollutants, leading to their presence in the effluent or sewage sludge. Consequently, these residues can enter the terrestrial environment through various routes, such as irrigation with treated wastewater, application of inadequately treated animal manure, or the use of biosolids in agriculture within the circular economy [1–3]. The dispersion of these emerging organic contaminants has far-reaching consequences, including adverse effects on soil microbial communities, the emergence of antimicrobial resistance, contamination of the food chain through crop uptake, negative impacts on crop yields (seed germination, biomass growth), and potential degradation of soil quality owing to detrimental effects on soil organisms. Additionally, these xenobiotics may undergo transformation into metabolites within plants or soil organisms. Given the limited understanding of these metabolic pathways, not all drug-related metabolites have been identified [2–7].

Past studies on xenobiotics have revealed that plants and soil organisms possess the capability to metabolize man-made chemicals through phases I and II. During phase I reactions, the parent compound (or its metabolite) undergoes transformative processes, such as hydrolysis, reduction, or oxidation, enhancing its reactivity and polarity. This enhancement involves the addition of functional groups such as -OH, -NH_2_, or -SH [1]. During phase II metabolism, xenobiotics undergo conjugation with endogenous molecules, such as acetyl, methyl, and sulfate groups, glucuronic acid, glucose, glutathione, glucopyranosyloxy and malonyl groups, pterin, methyl salicylate, leucyl, glutamic acid, glutamine, and various sugars and amino acids [4, 7–11]. Moreover, in plants, the occurrence of phase III metabolism, often referred to as the “green liver concept,” is notable. During this phase, conjugates have the ability to bind to insoluble components, such as lignin and polysaccharides, or undergo storage within cell vacuoles, a process known as sequestration [1, 4, 7]. Sequestration of pharmaceutical conjugates may offer an additional mechanism of bioaccumulation, extending beyond passive partitioning into lipids and other phases [1]. Consequently, the metabolism of plants and soil organisms holds the potential for an “endless” number of drug-related metabolites. This is due to the fact that these reactions, their combinations, and the resulting metabolites of parent substances remain inadequately investigated [11]. Furthermore, studies emphasize the imperative to identify drug-related metabolites and quantify them alongside parent substances in the environment. This is crucial for a comprehensive assessment of the environmental and potential health risks associated with the dissemination of pharmaceuticals across different environmental compartments [1, 10]. Pharmaceutical phytotoxic effects can lead to lower germination rates, reduced root and shoot mass, decreased reproductive rates, chlorosis, oxidative stress, tissue deformation, and altered enzymatic activity. The specific symptoms depend on factors such as contamination concentration, the type of pharmaceutical or metabolite, and their therapeutic class [12, 13]. The potential health risks to humans from environmental pharmaceutical residues arise from the unknown adverse effects of long-term exposure to trace amounts of these substances through the contaminated food chain [14]. While some studies [15, 16] have reported minimal health risks from consuming contaminated crops, the research on human risk and exposure remains premature due to a lack of comprehensive studies. Most PhAC metabolites have not been identified or included in these risk assessments. Additionally, these residues can contribute to the spread of antimicrobial resistance within both environmental ecosystems and human populations [17].

Recent studies [4, 7–9, 11, 18–22] addressing the identification of metabolites (Met-ID) typically employ cell cultures or already pregrown plants for uptake experiments under hydroponic conditions. However, this approach fails to fully mimic real growing conditions. Lettuce (*Lactuca sativa*) is the most commonly used plant in these experiments, although other plant species such as tomato, cucumber, black pepper, onion, maize, amaranth, rice, and pea have also been used. In these experiments, plants were exposed to non-relevant concentrations, ranging from 50 to 1000 times higher than the environmental concentrations, to facilitate the elucidation of the metabolite structure. Consequently, a substantial number of pharmaceutical metabolites are frequently detected. It is noteworthy that these elevated concentrations may induce the formation of metabolites not present at environmentally relevant levels due to their influence on plant metabolism. Commonly tested substances include ibuprofen, diclofenac, trimethoprim, naproxen, triclocarban, and carbamazepine, with contamination typically performed using a single substance per exposure experiment. Plant samples, both contaminated and non-contaminated, were collected at varying exposure intervals, determined by the experimental setup or the plant’s growth rate, spanning from hours to weeks. Root and leaf samples are extracted separately, and liquid chromatography coupled to high-resolution mass spectrometry is the prevailing analytical method. In the initial stages, a literature search for known metabolites is usually conducted, followed by the utilization of commercially available software from mass spectrometry vendors for data processing and identification of these metabolites [4, 7–9, 11, 18–22].

The primary objective of this study was to identify drug-related metabolites in earthworms and lettuce cultivated in both soil and a hydroponic environment after exposure to pharmaceutical contamination. The novelty of this study lies in the conduct of exposure experiments with a mixture of pharmaceuticals at environmentally more relevant concentrations than those in other studies. To achieve this, earthworms and lettuces were subjected to a mixture of six commonly used pharmaceuticals, namely atenolol, enrofloxacin, erythromycin, ketoprofen, sulfamethoxazole, and tetracycline — each representing a distinct pharmaceutical class. Moreover, the design of the study ensured that lettuce was exposed to contamination for almost the entire growth period. A novel study workflow for Met-ID was introduced, as described in detail in the “Met-ID study workflow” section of the manuscript. In contrast to certain vendor software, our study employed freely available software with available algorithms, enabling scientific scrutiny and validation of the process: (1) metabolite prediction, (2) in silico spectral library prediction, (3) data processing, and (4) statistical data evaluation. Specifically, our workflow integrated a triplet approach for metabolite structure prediction (combining software prediction, literature search, and common metabolic pathways) with the in silico prediction of MS^2^ spectra, a methodology not previously explored in the literature. Additionally, LC-qTOF measurements were conducted in both ESI + and ESI − modes, utilizing both data-dependent acquisition (DDA) and data-independent acquisition (DIA) modes to compare their efficiency in Met-ID.

## Materials and methods

### Chemicals

Ammonium nitrate (> 99.5%), calcium nitrate tetrahydrate (> 99%), citric acid monohydrate (≥ 99%), disodium hydrogen phosphate dodecahydrate (≥ 99%), ethylenediamine tetraacetic acid (EDTA, ≥ 99%), ferrous sulfate heptahydrate (> 99.7%), hydrochloric acid (35%), potassium nitrate (> 99.9%), sodium sulfate anhydrous (≥ 95%), and sulfuric acid (≥ 96%) were purchased from Lach:ner (Czech Republic). Acetonitrile (LC–MS grade), diethylenetriaminepentaacetic acid (≥ 99%), and methanol (LC–MS grade) were purchased from VWR (Czech Republic). Copper sulfate pentahydrate (> 98%), formic acid (LC–MS grade), manganese sulfate monohydrate (> 99%), sodium chloride (> 99%), sodium molybdate dihydrate (> 99.5%), and zinc sulfate heptahydrate (> 99%) were purchased from Sigma-Aldrich (Germany). Sodium hydroxide (> 98%) and potassium hydroxide (> 98%) were purchased from Penta Chemicals (Czech Republic). Boric acid (> 99.5%), magnesium sulfate heptahydrate (> 99%), and potassium phosphate monobasic (> 99.5%) were purchased from Lachema n.p. (Czech Republic).

The following pharmaceuticals (physicochemical properties in Table S1) were purchased from Sigma-Aldrich (Germany): atenolol (≥ 98%), enrofloxacin (≥ 99%), erythromycin (≥ 97%), ketoprofen (≥ 98%), sulfamethoxazole (≥ 98%), and tetracycline (≥ 98%). Individual stock standard solutions for the target pharmaceuticals were prepared in methanol at a concentration of 1 mg∙mL^−1^. A standard solution mixture containing all the target compounds was prepared by diluting the individual stock solutions in methanol to achieve a final concentration of 100 μg∙mL^−1^. This mixture was subsequently used for spiking in the uptake experiments and for LC–MS standard preparation.

Nylon syringe filters (13 mm, 0.22 μm) were purchased from Chromservis (Czech Republic). For QuEChERS, dispersive SPE (dSPE): DSC-18 SPE and PSA SPE were purchased from Sigma-Aldrich (Germany).

### Met-ID study workflow

**Fig. 1:**
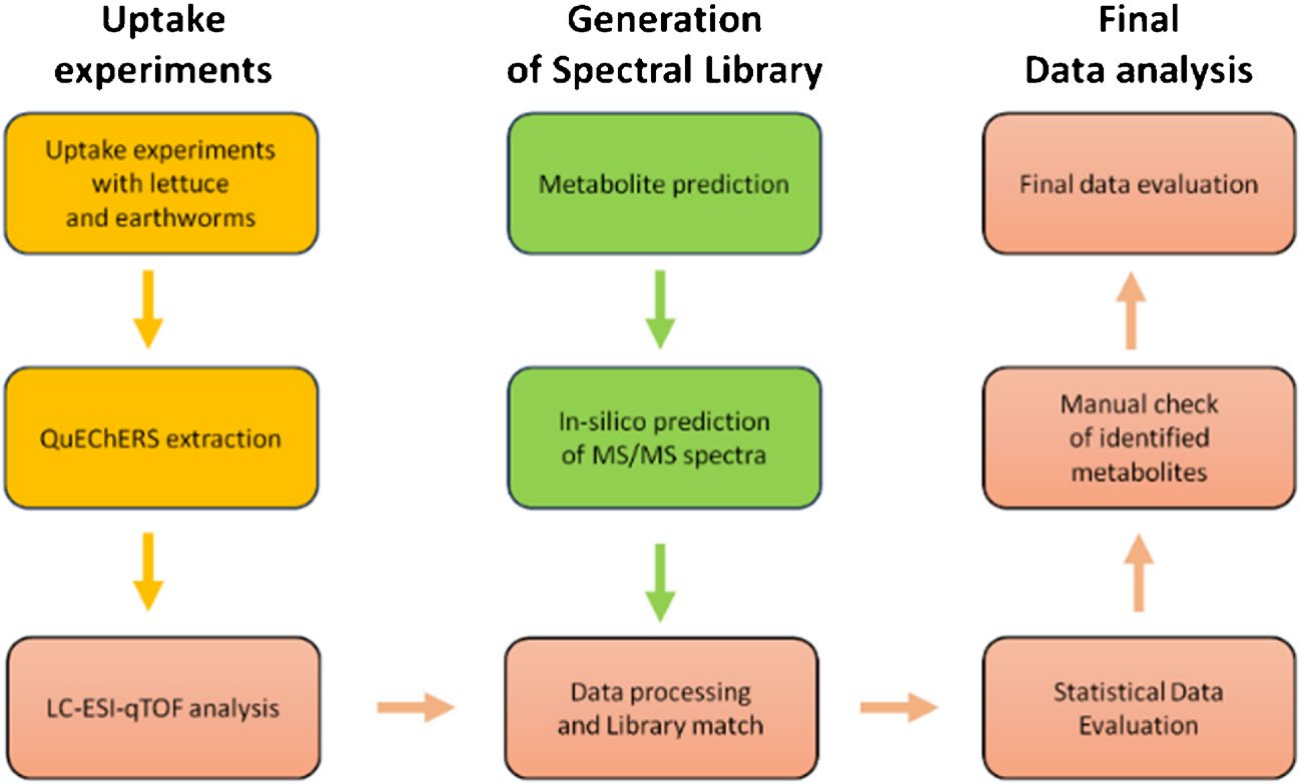
Met-ID study workflow

The outlined workflow (Fig. 1) was carefully devised and adhered to throughout this study. Initially, earthworm (*Eisenia fetida*) and lettuce (*Lactuca sativa*) uptake experiments were conducted, followed by QuEChERS extractions. Subsequent LC-ESI-qTOF analyses were performed in both positive and negative ionization modes, utilizing both DDA and DIA modes to assess their efficacy in feature detection. Prior to data processing in MS-DIAL 4.0 software, potential metabolites were predicted through a threefold approach: (1) literature search [20, 23–48]; (2) software prediction using BioTransformer 3.0 [49], GLORYx [50], and enviPath [51]; and (3) common metabolic pathways [4, 8, 9, 18, 20, 21, 36, 52–59]. Following this, an in silico MS/MS library for these compounds was generated using CFM-ID 4.0 [60] for both ESI + and ESI − . These libraries played a crucial role in the subsequent data processing stages within MS-DIAL 4.0 [61]. Finally, the acquired data were exported and subjected to statistical evaluation using MetaboAnalyst 4.0 [62] and subsequently reported.

### Description of uptake experiments

Lettuce was cultivated and grown under both hydroponic conditions and in soil environments. In both scenarios, the aquatic or soil environment was contaminated from the start of the experiment with a mixture of six pharmaceuticals (atenolol, enrofloxacin, erythromycin, ketoprofen, sulfamethoxazole, and tetracycline), each at a concentration of 10 μg∙L^−1^ (in hydroponics) or 10 μg∙g^−1^ (in soil). Concurrently, earthworms were exposed to the same pharmaceutical contamination in the soil environment as the lettuce. Detailed procedures for these uptake experiments are provided in the Supplementary Information—General, Appendix 1.

### QuEChERS sample extraction

Both the lettuce leaves, lettuce roots, and earthworms were extracted separately using our own previously validated and published method [63], detailed in the Supplementary Information—General, Appendix 2.

### Untargeted analysis using LC-ESI-qTOF

QuEChERS extracts were analyzed by ultra-high-performance liquid chromatography (Shimadzu Nexera X3, Japan) in tandem with quadrupole time of flight (Shimadzu LCMS-9050, Japan) with electrospray ionization (ESI). Chromatographic separation was achieved using a Shim-pack Velox C18 (100 × 2.1 mm, 1.8 µm) column at a flow rate 0.4 mL∙min^−1^ and temperature of 40 °C. The mobile phases consisted of (A) 0.01% formic acid (FA) in Milli-Q water and (B) MeOH. The elution gradient was 0–1 min, 95% A; 13–14 min, 0% A; and 14–18 min, 95% A. The injection volume was 4 µL for all samples. The qTOF operating parameters were set as follows: interface voltage, 1500 V; interface temperature, 300 °C; desolvation temperature, 250 °C; DL temperature, 250 °C; heat block temperature, 400 °C; nebulizing gas flow, 3 L∙min^−1^; heating gas flow, 10 L∙min^−1^; and drying gas flow, 10 L∙min^−1^. For data-dependent analysis (DDA), full scan MS (centroid mode, m/z 60–800, 50 ms scan time) were acquired followed by MS/MS mass scan (20 of dependent events, 20 ms scan time, CE spread 30 ± 25 eV) using precursor priority list containing predicted drug-related metabolites with a total of 0.45 s cycle time. For data-independent analysis (DIA), full scan MS (centroid mode, m/z 60–800, 50 ms scan time) were acquired followed by MS/MS mass scans (23 DIA MS/MS scans with a precursor isolation width of 32 Da, 22 ms scan time, CE spread 30 ± 25 eV) resulting in 0.56 s cycle time. For untargeted analysis, DDA + , DDA − , DIA + , and DIA − modes were employed to analyze the QuEChERS extracts. Mass accuracy correction was achieved using reference masses (m/z 118.086255; 322.048121; 622.028960) after each LC–MS run as external standard, aiming for a measurement accuracy of < 5 ppm. Data stability, reliability, and high quality were ensured through the injection of pooled quality control (pooled QC) at the beginning of the sequence (8 × times), after every fifth sample during the sequence, and samples were analyzed in a random order. Pooled QC was prepared by combining aliquots of the tested samples (both treated and untreated), following common practices in metabolomics studies.

### Prediction of pharmaceutical metabolites

To obtain the highest possible number of potential metabolites, the prediction of pharmaceutical metabolites involved the integration of various methods to generate an extensive list of potential drug-related metabolites. Three approaches were employed: (1) literature search, (2) software prediction, and (3) common metabolic pathways (reactions). To compile a comprehensive list of potential drug-related metabolites, a meticulous literature search was conducted. Metabolites from the following publications [20, 23–48] were extracted and documented in the compounds list within Supplementary Information – List of Predicted Metabolites – Sheet literature search. To predict potential metabolites, three distinct software tools were employed: BioTransformer 3.0 [49], GLORYx [50], and enviPath [51]. This comprehensive software suite was utilized to predict metabolites based on the chemical structures of parent drugs, using their SMILES codes. The resulting lists of potential metabolites generated by each software tool can be found in Supplementary Information – List of Predicted Metabolites – Sheet software prediction. Additionally, a comprehensive set of common metabolic pathways (reactions), as mentioned in previous studies [4, 8, 9, 18, 20, 21, 36, 52–59] or their combinations, was integrated to create a comprehensive list of potential metabolites. This systematic approach, detailed in Supplementary Information – List of Predicted Metabolites – Sheet List of common metabolic pathways, involved the application of a total of 144 different reaction pathways for each pharmaceutical.

### In silico prediction of MS/MS spectra

In the application of methods 1 (literature search) and 2 (software prediction) to generate compound lists, the known structures of metabolites facilitated the retrieval of their chemical formulas, molecular weights, and SMILES codes. However, when employing method 3 (common metabolic pathways/reactions), only the chemical formula and molecular weight were obtained. Because of the inability to predict the site of metabolism (SOM), generating precise structures, and subsequently SMILES codes, was not feasible because of the lack of freely available software solutions. For compounds with available precise structures (SMILES codes), a custom script was employed (Supplementary Information – Script for MS2 in silico prediction). CFM-ID 4.0 [60] was utilized along with the script to generate MS/MS spectra in both positive and negative ionization modes. CFM-ID produced product spectra at CE values of 10, 20, and 40 eV. The script processed the compound table input (Supplementary Information– chemical_list for All of the Metabolites and Supplementary Information – metabolites_R for metabolites predicted using common metabolic pathways) to create a spectral library, combining CE values of 10 and 20 eV to simulate the use of CE spread during DIA and DDA analyses. CE 40 eV was omitted to avoid the presence of low molecular fragments that could lead to false positives during library matching, lacking specific structural information. In the case of common metabolic pathways, the lack of precise chemical structures (SMILES codes) was effectively addressed as the script generated an MS/MS spectral library for the parent substance, including precursor ions [M + H]^+^ or [M-H]^−^ based on the monoisotopic molecular weight of the metabolite. To illustrate this process, consider the conjugation of sulfamethoxazole with glucose (Sulfamethoxazole-R63). The product ions predicted in silico for sulfamethoxazole are of m/z: 92.04948, 94.06513, and 156.01138, with the precursor ion [M + H]^+^ 254.05994. Consequently, the predicted MS^2^ spectrum for Sulfamethoxazole-R63 is 92.04948, 94.06513, 156.01138, and 254.09539, respectively, with the precursor ion [M + H]^+^ 416.11276 (Δm/z 162.0528). The finalized spectral library for the positive ionization mode can be accessed at Supplementary Information—Library Metabolites ESI + , whereas the negative mode library is available at Supplementary Information—Library Metabolites ESI-.

### Data processing in MS-DIAL

Following LC-qTOF analysis, the acquired data were converted into centroid mzML format. The converted data were then imported into MS-DIAL 4.0 [61], along with an in silico spectral library created in the MSP format. Within MS-DIAL, default settings were predominantly applied, with exceptions on tab identification (accurate mass tolerance in MS^1^, 1 mDa; and accurate mass tolerance in MS^2^, 5 mDa), and tab alignment (retention time tolerance 0.1 min). The processed data were subsequently exported for further analysis.

### Statistical analysis

The exported data from MS-DIAL 4.0 underwent pre-filtering based on specific criteria, in accordance with established guidelines [64–67]: (1) QC %RSD peak area for a particular feature must vary < 30%; (2) the feature must be present in QCs in at least > 50%; and (3) the feature must be present in any group at least > 70%. Subsequently, the data were imported into MetaboAnalyst 4.0 [62] for statistical analysis. Principal component analysis (PCA) was carried out to determine whether there were statistically significant differences between treated and control groups within each of the studied categories: earthworms and lettuce (leaves and roots). Volcano plot analysis was performed to identify features responsible for these significant differences (*p* < 0.05 and fold change (FC) 3.0). Finally, these statistically significant features were cross-referenced with our predicted spectral library and reported.

## Results and discussion

### Metabolic impact of pharmaceutical exposure

Similar to findings in environmental studies [68–72], results of PCA (Figs. S1–S3) illustrate whether statistically significant differences exist between non-contaminated and contaminated samples for both earthworms and lettuce. The most pronounced differences between contaminated and non-contaminated samples are observed in lettuce roots (Fig. S2), where the sample groups are distinctly separated. This is followed by lettuce leaves (Fig. S3), while in the case of earthworm samples, the groups are not clearly separated (Fig. S1); this does not imply the absence of differences; rather, it may be due to the limitations of PCA in capturing subtle variations in this dataset. Notably, most of the significant features underlying the differences between sample groups are linked to alterations in the metabolomes of earthworms and lettuce, involving a diverse range of compounds, including lipids, amino acids, saccharides, and others. Although these changes are pertinent to the overall understanding of the system, they fall outside the scope of this manuscript, which focuses on pharmaceutical metabolites. Furthermore, the assurance of high data quality and reliability in various measuring modes is reinforced by the close proximity and tight clustering of the QC pooled samples. Additionally, the graphical placement of QC samples within the figures, interspersed between different sample types, further contributes to the robustness of the data validation process.

In the process of feature picking, a volcano plot analysis was employed with parameters (*p* < 0.05 and positive fold change (FC > 3.0)). The use of positive fold change was deliberate, aligning with the focus on identifying only drug-related metabolites, that should not be in blank samples. As in previous studies [7, 19], the FC value was set at 3, mirroring the definition of the limit of detection (LoD), and low concentrations of substances. Subsequently, only features deemed statistically significant and showing a positive fold change were subjected to further evaluation (the quantity of significant features in Table S5). These significant features were cross-referenced with our in silico spectral library, which was designed for the matching of pharmaceutical metabolites. A total of 26 drug-related metabolites were successfully identified (Table 1, respectively Tables S6–S7 with SMILES codes).

**Table 1:** Presence of parent drugs and their metabolites in samples of earthworms and lettuce (roots and leaves) at different exposure times (*SP* software prediction, *LS* literature search, *CMP* common metabolic pathways)

Compound name	Method for metabolite prediction	Soil earthworms						Soil lettuce						Hydroponics lettuce						Reported in literature
		Soil	EW					Soil	Lettuce roots			Lettuce leaves		Water	Lettuce roots			Lettuce leaves		
		21 days	1 day	3 days	7 days	14 days	21 days	28 days	14 days	21 days	28 days	21 days	28 days	28 days	14 days	21 days	28 days	21 days	28 days	
Atenolol	-	✔	✔	✔	✔			✔						✔	✔	✔	✔	✔	✔	-
Atenolol-LS1	SP/LS/CMP		✔	✔	✔	✔	✔		✔	✔	✔	✔	✔	✔	✔	✔	✔			[23]
Enrofloxacin	-	✔	✔	✔	✔	✔	✔	✔	✔			✔	✔	✔	✔	✔	✔	✔	✔	-
Enrofloxacin-R63	CMP														✔	✔	✔			-
Enrofloxacin-R13	CMP														✔	✔	✔			-
Ciprofloxacin	SP/LS														✔	✔	✔			[73]
Enrofloxacin-R2	CMP														✔	✔	✔			-
Enrofloxacin-M242	SP														✔	✔	✔			-
Erythromycin	-	✔	✔	✔	✔	✔	✔	✔						✔	✔	✔	✔			-
Erythromycin-M1	SP/CMP													✔	✔					-
Erythromycin-M46	SP	✔												✔	✔	✔	✔			-
Erythromycin-M361	SP													✔	✔					-
Ketoprofen	-	✔	✔	✔	✔	✔	✔	✔	✔	✔	✔	✔	✔	✔	✔	✔	✔			-
Ketoprofen-R13	CMP								✔	✔	✔	✔	✔		✔	✔	✔			-
Ketoprofen-R23	CMP									✔	✔				✔	✔	✔			-
Ketoprofen-R63	LS/CMP									✔	✔				✔	✔	✔			[36]
Ketoprofen-R81	CMP								✔	✔	✔	✔	✔		✔	✔	✔			-
Ketoprofen-R87	LS/CMP									✔	✔									[36]
Ketoprofen-R93	LS/CMP									✔	✔									[36]
Ketoprofen-R96	CMP										✔									-
Ketoprofen-R111	CMP														✔	✔	✔			-
Ketoprofen-LS24	SP/LS/CMP									✔	✔				✔	✔	✔			[38]
Ketoprofen-LS25	SP/LS														✔	✔	✔			[38]
Ketoprofen-M648	SP									✔	✔									-
Ketoprofen-M835	SP								✔	✔	✔	✔	✔		✔	✔	✔			-
Sulfamethoxazole	-	✔	✔	✔	✔	✔	✔	✔	✔	✔	✔	✔	✔	✔	✔	✔	✔	✔		-
Sulfamethoxazole-LS1	SP/LS/CMP		✔	✔	✔	✔	✔		✔	✔	✔	✔	✔		✔	✔	✔			[39]
Sulfamethoxazole-LS6	LS														✔	✔	✔			[41]
Sulfamethoxazole-R63	LS/CMP								✔	✔	✔	✔	✔		✔	✔	✔			[41]
Tetracycline	-	✔	✔	✔	✔	✔	✔	✔	✔			✔		✔	✔	✔	✔			-
Tetracycline-LS2	LS														✔	✔	✔			[45]
Tetracycline-LS3	LS														✔	✔	✔			[45]

### Impact of prediction methods on metabolite identification

The prediction process identified a total of 3762 metabolites using three different approaches: 2704 metabolites were identified through software prediction (SP), 194 metabolites were discovered via literature search (LS), and 864 metabolites were identified using common metabolic pathways (CMP). After conducting the predictions, it became apparent that almost 90% of the predicted metabolites exhibited m/z values lower than 800 (3387 out of 3762). Hence, this threshold was utilized as the upper limit for LC-qTOF analysis to optimize the scan time and enhance the sensitivity of the measurements.

**Fig. 2:**
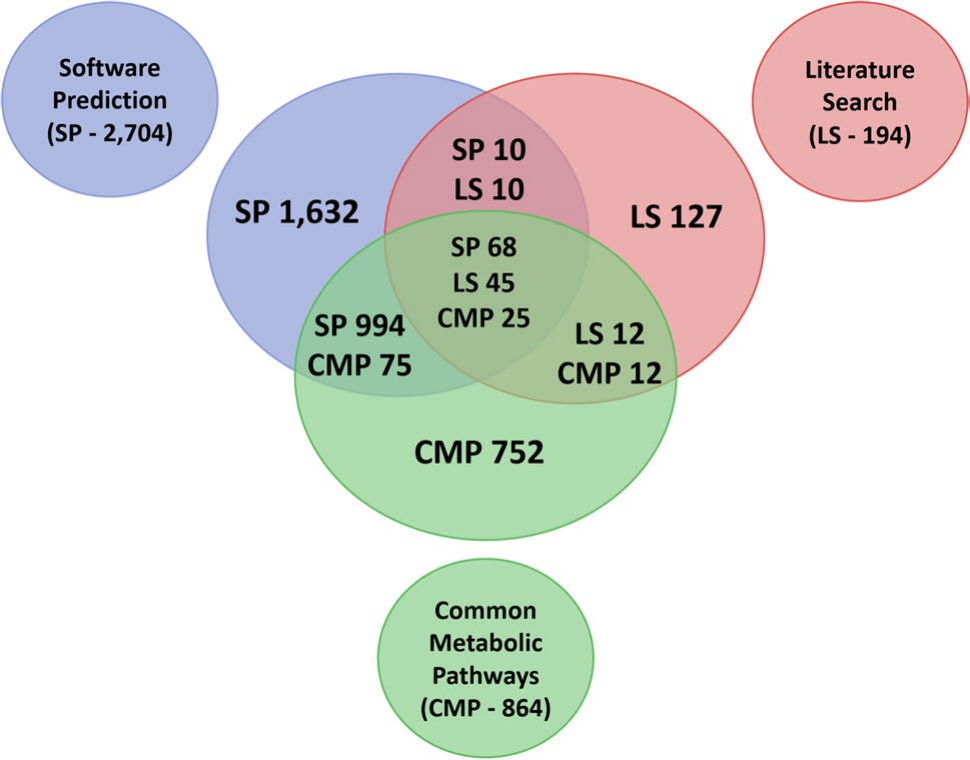
Venn diagram illustrating the overlap among three approaches for metabolite structure prediction, highlighting the number of metabolites predicted by each method

The overlap between the three prediction approaches is evaluated in Fig. 2 using a Venn diagram. Software predictions and literature searches identify exact molecular structures, treating structural isomers as distinct metabolites. In contrast, predictions based on common metabolic pathways provide only tentative structures, without differentiating between structural isomers. To accurately compare the approaches, several structural isomers were often grouped together under a single common metabolic pathway (reaction), as illustrated in Fig. 2. For example, when all three approaches overlapped, the software prediction identified 68 structures, while the literature search yielded 45 structures. All of these metabolites were accounted for by 25 common reaction pathways, due to the generation of structural isomers. This grouping was necessary to ensure a representative and meaningful comparison. The prediction of exact structures through SP can simplify metabolite identification, as it allows for the generation of more precise in silico MS^2^ spectra, unlike CMP, which only provides tentative structures. Furthermore, the partial overlap between CMP and SP (or also LS) suggests that the available software for metabolite prediction is not limited to simple oxidation, reduction reactions, hydrolysis, or conjugation with amino acids, saccharides, etc. It also predicts the cleavage of parent compounds into smaller molecules, which are not described by the list of common metabolic pathways, although sometimes it can be analytically quite challenging to link these small degradation product to specific small-drug molecules, as these compounds may also naturally occur within the organism. Nevertheless, the software prediction did not encompass all the common metabolic pathways described in the available literature [4, 8, 9, 18, 20, 21, 36, 52–59], contrary to our expectations.

This weak overlap may be due to several factors. The software’s prediction algorithms might be optimized for specific types of reactions or tailored to particular organisms, leading to the omission of pathways described in the literature for different organisms. Additionally, the complexity and variability of biological systems could result in metabolites that are not predicted by the software, even though they are reported in the literature. The algorithms might also focus on common metabolic routes, potentially missing less frequent or more complex pathways. Lastly, there may be a lag in updating prediction models with the latest scientific discoveries.

**Fig. 3:**
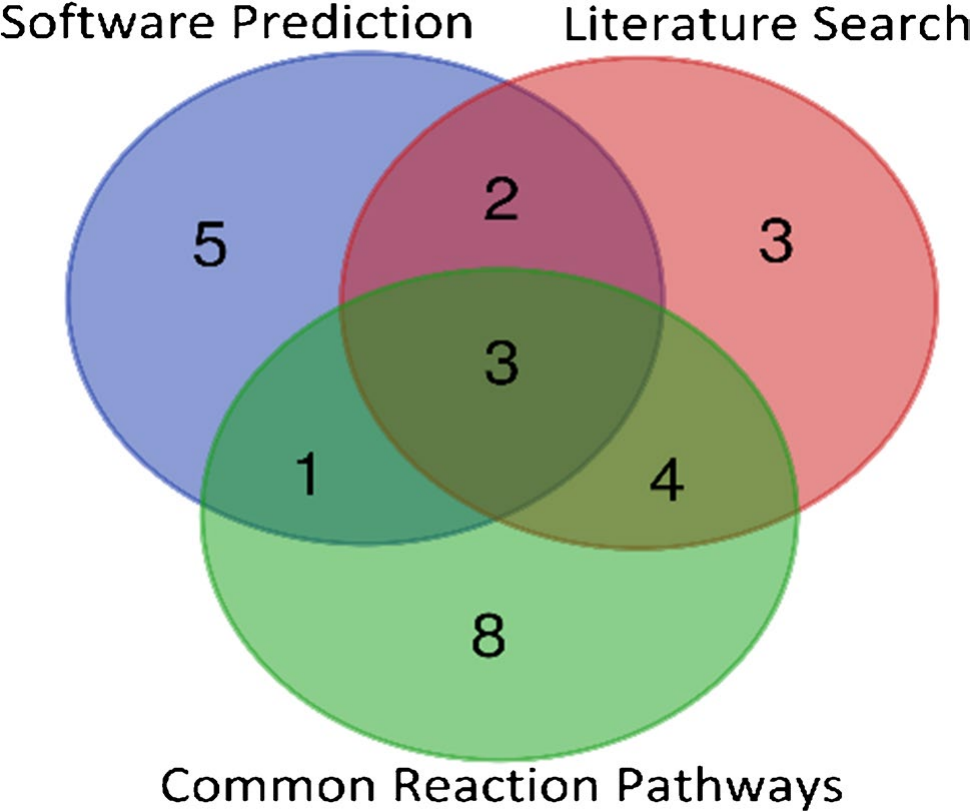
Venn diagram comparing metabolite identification efficiency across different prediction methods, showing the number of metabolites identified by each method

Following successful metabolite identification, a comparison of the different prediction methods for Met-ID success was visualized in a Venn diagram (Fig. 3 and Table S7). Breaking down the successful identifications, software prediction identified 11 compounds, literature search identified 12 compounds, and common reaction pathways identified 16 compounds. Notably, common reaction pathways resulted in a slightly higher number of successfully identified metabolites. However, each method produced a unique set of compounds, and only 3 of 26 substances were predicted by all three approaches. Given that no single approach demonstrated superiority, it can be concluded that the strategy employing all three methods for prediction was successful. Meanwhile, studies [7, 11, 13, 18–22, 53, 56, 74, 75] rely on either literature search, common metabolic pathways, or a simple blank subtraction in mass spectrometry. The comprehensive use of diverse prediction methods enhances the likelihood of capturing a broader spectrum of metabolites, thus contributing to a more thorough and reliable Met-ID process. Moreover, the overlap between the three approaches for successful metabolite identification (Fig. 3) is quite low, much like the overlap observed in the number of metabolites predicted by each method (Fig. 2). If we examine whether the most intense metabolites were identified by the SP, LS, or CMP approaches, we find that in the case of earthworms (Fig. S43), both metabolites (Atenolol-LS1 and Sulfamethoxazole-LS1) were predicted by all three approaches. In contrast, for *L. sativa* major metabolites (Figs. S44–S52), the major metabolites (such as Ketoprofen-R87, Ketoprofen-R81, Ketoprofen-R63, Sulfamethoxazole-R63, Enrofloxacin-R63) are formed through glucosidation, a process described in common metabolic pathways, but are not predicted by software predictions. Conversely, metabolites identified solely by SP (such as Enrofloxacin-M242, Erythromycin-M46, Erythromycin-M361, Ketoprofen-M648, and Ketoprofen-M835) exhibited lower ion intensities.

### Impact of MS/MS modes on metabolite identification

**Fig. 4:**
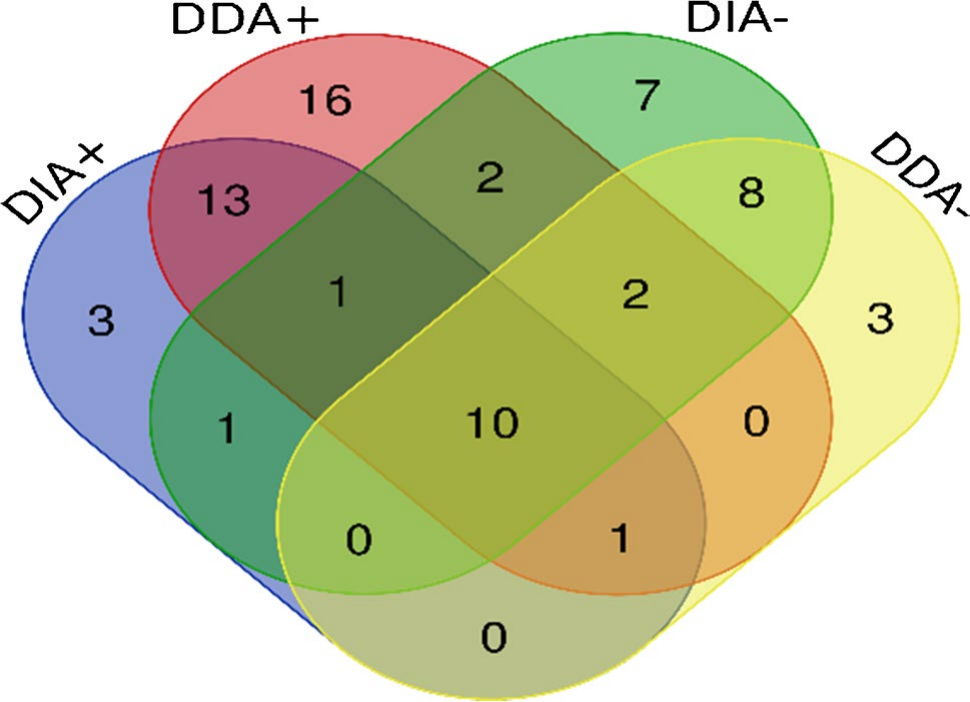
Venn diagram comparing metabolite identification efficiency across different MS/MS modes, showing the number of metabolites identified by each mode

In this analysis, various MS/MS modes were compared on the basis of the number of library matches, which were visualized using a Venn diagram (Fig. 4). Each metabolite:sample type combination was considered as one element, totaling 67 unique elements. ESI + demonstrated slightly more matches (74) than ESI − (55). We postulate that, despite the typical measurement of pharmaceuticals in ESI + , efficient ionization in ESI − was facilitated by conjugation with various endogenous molecules, such as glucose. Both ESI + and ESI − modes yielded both diverse and unique compounds in Met-ID, highlighting the efficiency of utilizing both modes and their complementarity. In ESI + , DDA + (16 unique metabolites) outperformed DIA + (3 unique metabolites), except in the case of earthworm samples, where both MS/MS modes successfully identified 2 pharmaceutical metabolites. This difference may be attributed to the use of a precursor ion list and better MS^2^ quality due to the low concentrations in the complex matrix. In ESI − , the difference between DIA − (7) and DDA − (3) was less significant, given the overall lower number of identified metabolites. Consequently, combining DDA + and DDA − resulted in 56 of 67 successful identifications while halving the analysis time. Previous studies on the identification of drug-related metabolites have commonly focused on ESI + [11, 13, 18–20, 22, 55, 56, 75], whereas in the case of ibuprofen [53] or triclocarban [74], ESI − is utilized. Rarely, both ionization modes are employed [7, 21]. MS/MS mode studies typically use either DDA [7, 11, 19] or DIA [18, 22, 74, 75] mode.

### Identification of pharmaceutical metabolites

The majority of environmental Met-ID studies [18, 19, 21, 22, 55, 56, 74] typically focus on the identification of metabolites derived from a single parent drug per experiment. To enhance the study throughput, we simultaneously investigated the metabolites of a mixture comprising six pharmaceuticals. In total, 26 drug-related metabolites were successfully identified (Table 1). The distribution among parent substances is as follows: atenolol (1), enrofloxacin (5), ketoprofen (12), sulfamethoxazole (3), tetracycline (2), and erythromycin (3). A pivotal study [76] characterized five identification confidence levels in HRMS for elucidating small molecules and their metabolites. These confidence levels are commonly employed in Met-ID studies [8, 11, 19, 22]. Some studies even synthesized metabolites after successful structure elucidation to attain level 1 confidence (confirmed structure by reference standard) [8, 19]. Within the scope of this study, only pharmaceutical metabolites with identification confidence level 1 (confirmed structure by reference standard), level 2 (probable structure by library spectrum match or experimental data), and level 3 (tentative structures supported with MS^2^ experimental data or, as in our case, by a library match with in silico MS/MS predictions) were reported. Metabolites with confidence levels 4 and 5 were excluded because of complex matrix, low concentrations, missing MS^2^ spectra, and overall low data reliability. The overall measured MS^2^ quality is influenced by the MS/MS measurement mode, with DDA spectral quality being higher than in the case of DIA due to differences in functionality. Additionally, sample matrix and metabolite concentration can negatively impact spectral quality [77]. In some instances, MS^2^ spectra for both parent drugs and metabolites were obtained in both ESI + and ESI − . All MS^2^ spectra of the identified metabolites in this study are illustrated in Figs. S4–S42, and their corresponding product ions are listed in Table S6.

For metabolites with known structures, the product ions were found to be in agreement with in silico predictions by CFM-ID 4.0 [60]. When the structure of a metabolite was already described, the observed product ions were consistent with available literature, such as acetyl-sulfamethoxazole (Fig. S6 with study [40]), conjugation of sulfamethoxazole with glucose (Fig. S8 with study [40]), conjugation of sulfamethoxazole with pterin (Fig. S10 with study [41]), atenolol acid (Fig. S12 with study [23]), conjugation of ketoprofen with glucose (Fig. S19 with study [36]), conjugation of ketoprofen with malonic acid and glucose (Fig. S21 with study [36]), and double conjugation of ketoprofen with glucose (Fig. S22 with study [36]).

In instances where only the tentative structure was known, derived from the prediction of the metabolite through common metabolic pathways, the product ions typically exhibited alignment with those of the parent substances. This alignment often included the formation of [M + H]^+^ (or [M-H]^−^) ions of the parent substances as intense product ions for the metabolite, especially in cases of phase II metabolism involving conjugation with saccharides or amino acids [21]. Phase III processes may encompass the formation of secondary conjugations, such as malonyl, which serves as a signal for transport from the cytoplasm to the vacuole [19]. Regarding metabolism phase I reactions (e.g., hydroxylation, methylation), the presence of precursor ion of the parent drug in MS^2^ spectra of metabolites is less common due to fragmentation rules, which are dependent on molecular structure. Despite this, the product ions of parent substances are commonly shared with drug-related metabolites.

This observation aligns with a study [18] that found that diclofenac metabolites tend to cleave off parts of the molecule attached during conjugation metabolism, even at low collision energies. This suggests that the ion is fragmented to yield characteristic fragments while retaining information about the intact precursor. Similar fragmentation patterns were also noted in other studies [11, 18, 20, 36, 53, 55, 75] for different compounds such as diclofenac, trimethoprim, ibuprofen, clarithromycin, naproxen, and mefenamic acid. From these findings in both ionization modes, specific fragmentation patterns emerge, such as Δm/z 162.0528 for conjugation with glucose [18, 55]; Δm/z 176.0320 for conjugation with glucuronic acid [18]; Δm/z 86.0004 for conjugation with malonic acid; Δm/z 248.0532 for conjugation with glucose and malonic acid [36]; or several multiples of Δm/z 162.0528 corresponding to the degree of conjugation with glucose. In the study [36], the glucosidation of mefenamic acid, naproxen, and ketoprofen was frequently observed, with glucosidation events occurring multiple times and accompanied by gradual losses of Δm/z 162.0528 in MS^2^ spectra. However, this phenomenon was not observed in this study, likely because of the lower concentrations. Various combinations of these conjugations, including those involving amino acids, and metabolic reactions from phase I can occur. Subsequent mass spectrometric losses resulting from these processes can be explored in detail in Supplementary Information – List of Predicted Metabolites – Sheet List of common metabolic pathways. The identified fragmentation pathways were exploited for the in silico prediction of MS^2^ spectra when the precise structure was not known. Additionally, it is important to note that, in the case of exposure involving a mixture of pharmaceuticals, caution should be exercised to avoid combining pharmaceuticals from the same group. This precaution is warranted because many pharmaceuticals within one group share identical product ions, making it challenging to reliably distinguish which metabolites belong to specific parent drugs.

In ESI + mode, atenolol and its metabolite share some of the following precursor and product ions: 267.1703, 250.1438, 249.1597, 225.1234, 208.0968, 190.0863, 178.0863, 134.06, 116.107, 98.0964, 74.0600, 72.0808, in agreement with studies [23, 78]. For sulfamethoxazole and its metabolite in ESI + , the common precursor and product ions are 254.0594, 156.0114, 108.04439, and 92.0495, consistent with study [41]. In the ESI − mode, the following ions were observed: 252.0448, 156.0125, and 92.0506. In ESI + mode, ketoprofen and its metabolite exhibit common precursor and product ions: 255.1016, 209.0961, 105.0335, as reported in article [34]. Additionally, in ESI − , ions 253.087 and 209.0972 were observed. For enrofloxacin and its metabolite in ESI + , the common precursor and product ions are 360.1718, 342.1612, 316.182, 245.1085, and 231.0928, in agreement with study [30]. In ESI + mode, tetracycline and its metabolite share the following precursor and product ions: 445.1606, 428.134, 410.1234, as reported in article [45], whereas in the ESI − mode, 443.146, 426.1194, 273.0768, and 271.0612 were observed. For erythromycin and its metabolite in ESI + , the common precursor and product ions are 734.4685, 716.4579, 576.3742, 558.3636, 540.3531, and 158.1176, as noted in study [79].

Additionally, during Met-ID, we considered the relative change of retention time (ΔRt). The ΔRt values were evaluated to align with the alterations in compound polarity. For instance, the ΔRt of Sulfamethoxazole-LS1 (acetyl-sulfamethoxazole) transitioned from 5.15 min to a higher Rt of 6.25 min, in agreement with study [40]. Similarly, the ΔRt of Sulfamethoxazole-R63 (glucose conjugation) shifted from 5.15 min to a lower Rt of 4.45 min, aligning with study [40]. Additionally, the ΔRt of Atenolol-LS1 (atenolol acid) moved from 2.94 min to a higher Rt of 4.22 min, as supported by study [23]; other ΔRt can be seen in Table S6.

### Metabolism of pharmaceuticals

The biotransformation of xenobiotics involves two distinct phases. In phase I of our study, processes such as oxidative deamination, de-alkylation, hydroxylation, or hydrolysis occur, leading to the inclusion or exposure of reactive and hydrophilic groups in the xenobiotic structures. Transitioning into phase II, xenobiotics or their phase I metabolites can undergo conjugation reactions with endogenous compounds such as glucose, malonic acid, or pterin [55]. The identified metabolites in *Eisenia fetida* and *Lactuca sativa* underwent a combination of reactions from both metabolism phases I and II, as summarized in Table S6. The distribution of metabolites among parent substances is as follows: atenolol (1), enrofloxacin (5), ketoprofen (12), sulfamethoxazole (3), tetracycline (2), and erythromycin (3). The overall metabolic fate of pharmaceuticals in plants and soil organisms can be influenced by several factors, including the chemical structure of the compound, soil properties, contamination levels, plant species, cells/whole organism, and environmental conditions [11, 19, 21, 55, 80]. The study [55] highlighted the differences in biotransformation between ribwort cell suspension and whole ribwort regenerants. Although both model systems exhibited similar types of biotransformation reactions, there were significant differences in the spectrum and abundance of the detected metabolites. Overall, existing studies [11, 19, 21, 55, 80] have reported a wide range of metabolites, diverse metabolic pathways within the same pharmaceutical class, varying ratios of parent drugs and metabolites, and concentration dependencies across different sampling times. Our findings align with the observation that different metabolic pathways were evident for various parent drugs, even when experiments involved simultaneous exposure to a mixture of these pharmaceuticals at the same concentration under identical experimental conditions. Despite conducting a thorough literature search for metabolites (Supplementary Information – List of Predicted Metabolites – Sheet literature search), our study revealed distinct metabolites, even for parent drugs previously investigated. For example, a study [41] reported formyl-SMX and methylsalicylate-SMX, which were not identified in our study. Some pharmaceuticals displayed a preference for conjugation with glucose (e.g., ketoprofen — 6 metabolites, enrofloxacin — 1, sulfamethoxazole — 1), whereas others exhibited cleavage reactions of functional groups (tetracycline — 2 metabolites) or saccharide groups (erythromycin — 2 metabolites), as summarized in Table S6. Notably, ketoprofen showed the highest number of drug-related metabolites (12) in plants, consistent with a previous study [36]. A noteworthy disparity in metabolism pathways between earthworms and lettuce lies in the considerably broader spectrum of detected metabolites in lettuce (27 metabolites, while only 2 in earthworms). This difference could be attributed to the different enzymes and also presence of phase III metabolism in plants, a process involving the storage of metabolites within cell vacuoles, commonly referred to as sequestration (green liver concept) [1, 4, 7]. Additionally, other studies have identified several intermediates of metabolic reactions [41, 44], whereas in our case, we only identified Ketoprofen-LS24 as an intermediate in the transformation to Ketoprofen-LS25, consistent with the findings of the study [38]. Moreover, analysis of the spatial distribution of both parent drugs and metabolites could shed light on their origin and whether they accumulate in specific parts of the body or organs.

### Occurrence of pharmaceuticals and their metabolites in the samples

Table 1 summarizes the presence of parent drugs and their metabolites in earthworm and lettuce samples at various exposure times: for earthworms (1, 3, 7, 14, and 21 days) and lettuce (14, 21, and 28 days). Table 1 also indicates whether the compounds were present in the soil or water environment at the end of the experiment, providing insights into where the metabolites were formed. The number of identified metabolites in different lettuce samples includes lettuce roots — hydroponics (22), lettuce leaves — hydroponics (0), lettuce roots — soil (13), lettuce leaves — soil (6), and earthworm tissue (2).

Among all the parent pharmaceuticals and their metabolites detected in our study, only a subset of erythromycin metabolites was identified in both aqueous and soil environments at the end of the exposure experiments. This contrasts with studies [9, 10], which generally report that metabolites of pharmaceuticals commonly form in the environment. The observed discrepancy may be due to differences in experimental design, including variations in the specific pharmaceutical tested (our study focused on atenolol, enrofloxacin, erythromycin, ketoprofen, sulfamethoxazole, and tetracycline), the pharmaceutical concentrations, exposure duration, physicochemical properties of the soil, the volume of soil and water, and environmental conditions such as temperature, humidity, and photoperiod. Additionally, differences in the presence and activity of microorganisms could also influence the formation and detection of metabolites.

Earthworm tissues contained all six parent pharmaceuticals (atenolol — ATE, enrofloxacin — ENR, ketoprofen — KPF, sulfamethoxazole — SMX, tetracycline — TC, and erythromycin — ERY). In contrast, lettuce roots and leaves from both hydroponic and soil environments did not contain all the parent substances. Surprisingly, despite the initial discrepancy, lettuce samples ultimately contained a significantly higher number of metabolites compared to earthworm tissues. Specifically, only two metabolites were detected in earthworm tissues: ATE-LS1 (atenolol acid [23]) and SMX-LS1 (acetyl-sulfamethoxazole [39, 40]). Both compounds, previously described in the literature, were consistently present in earthworm tissues throughout the experiment, spanning from day 1 to day 21, as illustrated by the normalized peak intensity displayed in Fig. S43.

In the lettuce roots under hydroponic conditions, the following metabolites of parent drugs, along with their normalized intensities, were consistently present across all samples taken on days 14, 21, and 28: atenolol (ATE-LS1; abundance in Fig. S44B), enrofloxacin (ciprofloxacin, ENR-R2, ENR-R13, ENR-R63, and ENR-M242; abundance in Fig. S48), ketoprofen (KPF-R13, KPF-R23, KPF-R63, KPF-R81, KPF-R111, KPF-LS24, KPF-LS25, and KPF-M835; abundance in Fig. S46), sulfamethoxazole (SMX-LS1, SMX-LS6, and SMX-R63; abundance in Fig. S47B), tetracycline (TC-LS2, TC-LS3; abundance in Fig. S49), and erythromycin (ERY-M1, ERY-M46, and ERY-M361; abundance in Fig. S50). All of these metabolites were present in all samples on days 14, 21, and 28, except for ERY-M1 and ERY-M361, which were only present on day 14. The three ERY metabolites were also detected in the aqueous medium, making it difficult to distinguish whether they were formed in both the aqueous environment and lettuce roots or only in the medium and subsequently taken up. In the lettuce roots under soil conditions, the following metabolites of parent drugs, along with their normalized intensities, were identified: atenolol (ATE-LS1; abundance in Fig. S44A), ketoprofen (KPF-R13, KPF-R23, KPF-R63, KPF-R81, KPF-R87, KPF-R93, KPF-R96, KPF-LS24, KPF-M648, and KPF-M835; abundance in Fig. S45), and sulfamethoxazole (SMX-LS1, and SMX-R63; abundance in Fig. S47A). Metabolites ATE-LS1, KPF-R13, KPF-R81, KPF-M835, SMX-LS1, and SMX-R63 were consistently present in all samples over the span of 14, 21, and 28 days. Metabolites KPF-R23 to KPF-R63, KPF-R87, KPF-R93, KPF-LS24, and KPF-M835 were in samples from day 21. KPF-R96 was only in samples taken on day 28. As indicated by our results, the formation of certain metabolites occurs later during the exposure experiment, influenced by whether lettuce is cultivated under hydroponic or soil conditions. This difference is notably caused the concentrations of parent drugs in the roots, thereby affecting the kinetic rate of metabolite formation. Additionally, these cultivation conditions can influence the ratios between parent drugs and both major and minor metabolites. In lettuce root samples, a higher number of metabolites (22) were detected under hydroponic conditions compared with the soil environment (13). Remarkably, in the soil environment, roots contained only metabolites of atenolol, ketoprofen, and sulfamethoxazole, excluding enrofloxacin, tetracycline, and erythromycin, despite the detection of their parent compounds in the roots. This discrepancy, even with roots distributed throughout the PET pot, is likely attributable to a combination of the physicochemical properties of pharmaceuticals (Table S1) and soil properties (Table S4). In agreement with previous studies [16, 81, 82], the lower uptake of these parent drugs can be attributed to significantly higher Kd values in soil (enrofloxacin 260–6310 L∙kg^−1^ [83], tetracycline 420–1030 L∙kg^−1^ [83], erythromycin 8.3–128 L∙kg^−1^ [83]), molecular weight, their pKa values, and soil properties, particularly organic matter content, pH value, and concentrations of divalent cations (such as Ca^2+^, Mg^2+^) [84, 85]. Under hydroponic conditions, where soil factors are excluded, only the physicochemical properties of the substances and plant metabolism influence uptake. This explains why the majority of studies [82, 86] employ hydroponic conditions to assess the impact of pharmaceutical properties on uptake.

After the uptake of the parent compound by lettuce roots, translocation to leaves may occur, either as the parent drug with potential subsequent metabolization or the translocation of metabolites from roots to leaves. Previous studies [19, 55, 74] have reported that roots contain significantly higher concentrations of pharmaceuticals than leaves. This finding aligns with the number of identified metabolites in lettuce roots (26) and leaves (6), as well as with the abundances in Figs. S44–S52. Furthermore, the concentration of pharmaceuticals in plants depends on the contamination level of the environment. The following metabolites were detected in lettuce leaves grown in a soil environment: atenolol metabolite (ATE-LS1), ketoprofen (KPF-R13, KPF-R63, KPF-M835; abundance in Fig. S51), and sulfamethoxazole (SMX-LS1 and SMX-R63; abundance in Fig. S52). All of these metabolites were also detected in lettuce roots. Interestingly, these metabolites were present only when lettuce was grown in soil, although the abundance of the parent compound and its metabolites was significantly higher under hydroponic conditions. Similar to lettuce roots, the highest number of metabolites was ketoprofen-related. According to previous studies [10, 19], the distribution of compounds in plants appears to be affected by the molecular size, pharmaceutical charge, and other physicochemical properties. However, the pH in different plant organelles (vacuole, xylem, phloem, and cytosol) may differ from each other and from the pH in soil or hydroponic solution. Therefore, compounds taken up easily do not necessarily distribute well within plants. The diverse pattern of metabolites in different vegetables indicates that the uptake and metabolism of micropollutants are plant-specific and difficult to predict.

## Conclusion

A novel workflow for high-throughput identification of pharmaceutical metabolites across various matrices, utilizing open-source software and LC-HRMS analyses, was developed and presented. This workflow featured a triplet approach for metabolite structure prediction — integrating software-based predictions, literature review, and known metabolic pathways — which led to the prediction of 3762 metabolites corresponding to six parent compounds: atenolol, enrofloxacin, erythromycin, ketoprofen, sulfamethoxazole, and tetracycline. In silico mass spectral libraries were generated for both positive and negative ionization modes and uploaded to MS-DIAL software for processing DDA and DIA measurements. Ultimately, 26 statistically significant metabolites (*p* < 0.05) were identified in *E. fetida* and *L. sativa*.

The main advantage of this novel workflow is its capacity for high-throughput data analysis, including statistical evaluation, using only freely available software compatible with instruments from all vendors. Additionally, it can be adapted for other organic micropollutants beyond pharmaceuticals by simply importing different chemical structures. However, the workflow has limitations due to the software’s inability to predict all potential metabolites, given the unique metabolic profiles of different organisms. In this study, these limitations were addressed through literature reviews and the incorporation of common metabolic pathways. Although, discrepancies between in silico mass spectra and experimentally obtained MS^2^ spectra may still occur, due to the dependence on available data, future improvements are anticipated. As more data becomes available and software evolves, prediction accuracy and metabolite coverage are expected to improve significantly. This advancement will enhance the estimation of environmental and health risks by enabling more accurate identification of metabolites.

Nevertheless, the implications of this study confirm significant concerns about environmental and health risks, particularly given the number and abundance of pharmaceutical metabolites relative to parent substances. Currently, only parent compounds are quantified in environmental and biological matrices, as these metabolites are often not identified, potentially leading to an underestimation of these risks. Pharmaceutical metabolites could contaminate the food chain, posing health risks from long-term dietary intake of trace concentrations, and may also contribute to the rise of antimicrobial resistance in the environment, potentially impacting human health. In the future, appropriate legislation should be implemented to limit the dissemination of both pharmaceutical and metabolite residues, as well as antibiotic-resistant genes into the soil environment. Since these contaminants can be removed from wastewater, animal manure, and biosolids more easily than from the soil environment using processes such as advanced oxidation and aerobic/anaerobic fermentation.

## Supplementary Information

Below is the link to the electronic supplementary material.

Supplementary file1 contains general and detailed information about Uptake experiments, QuEChERS extraction methods, modified Sonneveld’s recipe for the hydroponic solution; physicochemical properties of pharmaceuticals; physicochemical properties of soil; PCA of different samples (earthworms, lettuce roots, lettuce leaves); Overview of identified metabolites and their structure as SMILES code; Overview of the method of prediction of identified metabolites; MS2 spectra of all identified metabolites; and normalised abundances of parent pharmaceuticals and their metabolites. (DOCX 3784 KB)

Supplementary file2 consists of several files. File “List of Predicted Metabolites” contains list of all predicted metabolites from three different prediction methods (software prediction, literature search, common metabolic pathways) and List of common metabolic pathways. File “chemical_list” encompasses all metabolites, including those with precisely known structures with available SMILES and InChI, as well as metabolites predicted by common metabolic pathways where SMILES and InChI are not available. This file explicitly specifies for which metabolites the MS2 spectral library should be predicted and included in the output library. File “metabolites_R” contains metabolites predicted by common metabolic pathways, and its purpose is to generate the MS2 spectral library for these particular metabolites. File “Library Metabolites ESI +” is In silico predicted MS2 Library for pharmaceutical metabolites in ESI+ in msp format. File “Library Metabolites ESI-” is In silico predicted MS2 Library for pharmaceutical metabolites in ESI- in msp format. (7Z 906 KB)

Supplementary file3 presents the script for MS2 in silico prediction. Document outlines instructions for running the script, required input data, output details, and information about the generated MS/MS spectral library. (DOCX 23 KB)

## Data Availability

Data not included in the main text or SI can be accessed by contacting the corresponding author.
