# Supplementary material for: Pharmaceutical metabolite identification in lettuce (Lactuca sativa) and earthworms (Eisenia fetida) using liquid chromatography coupled to high-resolution mass spectrometry and in silico spectral library
Source: Anal Bioanal Chem. 2024 Sep 10;416(28):6291–306. doi: 10.1007/s00216-024-05515-2 (PMC11541386; doi:10.1007/s00216-024-05515-2)
Supplement: Supplementary file 1 — Supplementary file1 contains general and detailed information about Uptake experiments, QuEChERS extraction methods, modified Sonneveld’s recipe for the hydroponic solution; physicochemical properties of pharmaceuticals; physicochemical properties of soil; PCA of different samples (earthworms, lettuce roots, lettuce leaves); Overview of identified metabolites and their structure as SMILES code; Overview of the method of prediction of identified metabolites; MS2 spectra of all identified metabolites; and normalised abundances of parent pharmaceuticals and their metabolites. (DOCX 3784 KB) [file 216_2024_5515_MOESM1_ESM.docx]

**Supplementary Information for**

**Pharmaceutical Metabolite Identification in Lettuce (Lactuca sativa) and Earthworms (Eisenia fetida) Using Liquid Chromatography Coupled to High-Resolution Mass Spectrometry and In Silico Spectral Library**

Jan Fučík^1,*^, Stanislav Fučík^2^, Sascha Rexroth^3^, Marian Sedlář^4^, Helena Zlámalová Gargošová^1^, Ludmila Mravcová^1^

*^1^ Institute of Chemistry and Technology of Environmental Protection, Faculty of Chemistry, Brno University of Technology, Purkyňova 118, 612 00 Brno, Czech Republic*

*^2^* *Faculty of Electrical Engineering and Communication, Brno University of Technology, Technická 3058/10, 616 00 Brno, Czech Republic*

*^3^* *Shimadzu Europa GmbH, Albert-Hahn-Straße 6, 472 69 Duisburg, Germany*

*^4^* *CEITEC Brno University of Technology, Purkyňova 656/123, 612 00 Brno, Czech Republic*

*corresponding author: [*xcfucikj@vutbr.cz*](mailto:xcfucikj@vutbr.cz)

**Table of Contents**

**Appendix 1.** Description of Uptake Experiments

**Appendix 2.** QuEChERS sample extraction

**Table S1.** Modified Sonneveld's recipe for the hydroponic solution (tank A, Total Volume 2.5 L)

**Table S2.** Modified Sonneveld's recipe for the hydroponic solution (tank B, Total Volume 2.5 L)

**Table S3.** Physico-chemical properties of pharmaceuticals

**Table S4.** Sampling location and physico-chemical properties of soil

**Figure S1-S3.** PCA of different samples (earhworm, lettuce roots and leaves)

**Table S5.** The quantity of significant features in volcano plot (p<0.05, FC > 3) specific to individual sample types across various MS/MS modes

**Table S6.** Overview of identified metabolites in earthworms and lettuce samples by LC-qTOF

**Table S7.** Overview of the method of prediction for identified metabolites and their SMILES codes

**Figure S4-S42.** MS^2^ spectra of all identified metabolites

**Appendix 3.** Normalized abundances of metabolites

**Figure S43-S52.** Normalised abundances of parent pharmaceuticals and their metabolites

**This document contains in total:** 30 pages; 7 Tables, 52 Figures

**Appendix 1.** Description of Uptake Experiments

**Uptake of Pharmaceuticals by Lettuce under Hydroponic Conditions**

This study was conducted within a controlled environment using a grow box (Green-Qube 1020L) with a 16-h photoperiod (17,500 lux; LED panel: ViparSpectra XS2000 230W), maintaining an air temperature of 23±1°C and air humidity of 45±5%. To ensure proper air exchange and flow, a single extraction fan and two oscillating fans were strategically placed within the grow box. Hydroponic experiments were conducted utilizing the deep water culture (DWC) method to cultivate lettuce. Dark duct tape-wrapped aquariums (20x20x20 cm) served as containers. For the hydroponic solutions, salts according to a modified Sonneveld's recipe (Table S2-S3) for lettuce were dissolved in deionized water. This solution was then spiked with a mixture of six pharmaceuticals (Atenolol, Enrofloxacin, Erythromycin, Ketoprofen, Sulfamethoxazole, and Tetracycline), each at a concentration of 100 μg∙L^-1^. Lettuce seeds (Lactuca sativa – *Australischer Gelber*) were acquired from FloraSelf (Czech Republic). Pre-grown lettuces (5 days old) were then planted into rock wool and hydroponic cups, with four replicates for each experiment. Electrical conductivity (EC) adjustments were made throughout the experiment: 0.95-1.05 mS∙cm^-1^ up to the 14^th^ day, 1.3-1.5 mS∙cm^-1^ up to the 21^st^ day, and 1.8-1.9 mS∙cm^-1^ up to the 28^th^ day. The pH value was maintained at 6.3±0.1 for the entire duration, with daily adjustments. Water volume was replenished daily to maintain consistency in the uptake experiments. The aquariums were randomly positioned in the grow box, and their locations were rotated every third day to compensate for variations in light intensity. Aeration of the aquariums occurred for 10 min every hour. Lettuces were sampled after 14, 21, and 28 days of exposure, including both control and contaminated samples. Sampling involved two lettuces from each aquaria after 14 days, followed by a single lettuce every week. Leaves and roots obtained from the samples were washed in deionized water to remove pharmaceuticals from the plant surface. Water samples were collected at the start and end of the experiment.

**Uptake of Pharmaceuticals by Lettuce in Soil Environment**

This part of the study was conducted within a grow box, mirroring the conditions of the hydroponic experiments with lettuce. The soil was spiked with a mixture of six pharmaceuticals, each at a concentration of 10 μg∙g^-1^ dry weight (physicochemical properties of soil in Table S4). Each PET pot (diameter of 95 mm; height of 80 mm; without drainage) was filled with 500±1 g dry weight of soil. Subsequently, four pre-grown lettuces (5 days old) on cotton wool were planted in each pot, and four replicates of each experiment were established. Initially, dry soil was watered with tap water up to 40% maximum water holding capacity (MWHC) and was consistently watered daily to maintain constant soil humidity throughout the uptake experiments. Weekly applications of a water-soluble organic liquid fertilizer (Natura, Czech Republic) with N-P-K 6.4-1.7-9.0 were employed as recommended. The pots were arranged randomly within the grow box, with pot positions altered every third day to counterbalance variations in light intensity. Lettuces were sampled after 14, 21, and 28 days of exposure, encompassing both control and contaminated samples. Sampling involved three lettuces from each pot after 14 days and a single lettuce after 21 or 28 days. Samples of leaves and roots obtained were washed in deionized water to remove pharmaceuticals from the plant surface. Soil sampling occurred at the start and end of the experiment.

**Uptake of Pharmaceuticals by Earthworms in the Soil Environment**

Earthworms (*Eisenia fetida*) were purchased from a local fish store called ProRyby (Czech Republic). The uptake experiment adhered to the Organisation for Economic Cooperation and Development (OECD) guidelines. Preceding the beginning of the experiment, the earthworms underwent a 3-day acclimatization period in uncontaminated soil. This phase was succeeded by a 1-day depuration period on filtration paper in the dark. The soil was spiked with a mixture of 6 pharmaceuticals, each at a concentration of 10 μg∙g^-1^ dw of soil. Subsequently, 50 ± 0.1 g of dry soil was carefully measured into each 100 mL beaker, and one earthworm per beaker was transferred into the soil. To facilitate proper aeration, the beakers were covered with perforated food plastic wrap. Soil humidity was adjusted to 40% MWHC, meticulously checked, and adjusted every third day. Throughout the 24-h light period, the experiment maintained a temperature of 20 ± 1 °C. Earthworms were provided with flakes on the first day of the experiment and subsequently every third day. Sampling occurred after 1, 3, and 5 days of exposure, with the depuration phase lasting 1 day. Post-depuration, the earthworms underwent lyophilization and extraction using the QuEChERS method. Soil sampling occurred both at the start and end of the experiment.

**Appendix 2.** QuEChERS Sample Extraction

**QuEChERS Extraction of Lettuce Samples**

The lettuce leaves and roots were extracted separately using our own already validated and published method [1]. Briefly, 0.1 g of lyophilized and homogenized lettuce leaves were weighed in a 50 ml PE centrifugation tube. Subsequently, ceramic homogenizers were added, followed by pipetting of 5 ml of extraction medium (MeOH:McIlvaine buffer pH 2.6:ACN in ratio 8:20:72). After that, the sample was vortexed for 1 min. Subsequently, separation salts (2g anhydrous Na_2_SO_4_ and 0.5g NaCl) were added, followed by vortexing for 1.5 min and centrifugation (3,500 rpm) for 10 min. After centrifugation, 2 ml of the organic phase was pipetted into a 15 ml PE centrifugation tube already containing dSPE sorbents (12.5 mg DSC-18, 12.5 mg PSA, and 225 mg of anhydrous Na_2_SO_4_). Subsequently, the sample was vortexed for 1 min and centrifuged (3,500 rpm) for another 10 min. Finally, the sample was filtered through 0.22 μm nylon syringe filters (diameter 13 mm) to a 2 ml glass vial.

**QuEChERS Extraction of Earthworm Samples**

The earthworms were extracted using our own already validated and published method [1]. Briefly, 0.1 g of lyophilized and homogenized earthworms was weighed in a 50 ml PE centrifugation tube. Subsequently, 10 mg of EDTA was weighed and ceramic homogenizers were added, followed by pipetting of 5 ml of extraction medium (MeOH:McIlvaine buffer pH 2.6 in a ratio 80:20). Then, the sample was vortexed for 1 min. Consequently, separation salts (2 g anhydrous Na_2_SO_4_ and 0.5 g NaCl) were added, followed by vortexing for 1.5 min and centrifugation (3,500 rpm) for 10 min. After centrifugation, 2 ml of the organic phase was pipetted into a 15 ml PE centrifugation tube already containing dSPE sorbents (12.5 mg DSC-18, 12.5 mg PSA, and 225 mg of anhydrous Na_2_SO_4_). Finally, the sample was vortexed for 1 min and centrifuged (3,500 rpm) for another 10 min. Finally, the sample was filtered through 0.22 μm nylon syringe filters (diameter 13 mm) to a 2-ml glass vial, followed by LC-MS analyses.

**Table S1** Physico-chemical properties of pharmaceuticals [2–4]; N.D. not determined

| **Pharmaceutical group** | **Substance name** | **Chemical formula** | **Mw [-]** | **pKa [-]** | **log P [-]** | **Kd [L∙kg^-1^]** | **Solubility in water [mg∙L^-1^]** |
| --- | --- | --- | --- | --- | --- | --- | --- |
| Beta Blocking Agents | Atenolol | C_14_H_22_N_2_O_3_ | 266.3 | 9.6 | 0.2 | 15 | 40 |
| Fluoroquinolones | Enrofloxacin | C_19_H_22_FN_3_O_3_ | 359.4 | 5.55; 7.24 | -0.2 | 260-6,310 | 53.9 |
| Macrolides | Erythromycin | C_37_H_67_NO_13_ | 733.9 | 8.9 | 2.7 | 8.3-128 | 4.2 |
| Antiinflammatory and Antirheumatic Products, Non-Steroids | Ketoprofen | C_16_H_14_O_3_ | 254.3 | 4.0 | 3.1 | 0.1-11 | 51 |
| Sulfonamides | Sulfamethoxazole | C_10_H_11_N_3_O_3_S | 253.3 | 1.6; 5.7 | 0.9 | 0.6-4.9 | 610 |
| Tetrayclines | Tetracycline | C_22_H_24_N_2_O_8_ | 444.4 | 3.3; 7.7 | -2 | 420-1,030 | 231 |

**Modified Sonneveld's recipe for the hydroponic solutions**

Note: Tank A and Tank B salts are mixed in ratio 1:1 to EC value as stated in Supplementary Information, Appendix 1.

**Table S2** Modified Sonneveld's recipe for the hydroponic solution (tank A, Total Volume 2.5 L) [5]

| **Salt** | **Weight [g]** |
| --- | --- |
| Ca(NO_3_)_2_·3H_2_O | 121.70 |
| NH_4_NO_3_ | 9.50 |
| KNO_3_ | 110.6 |
| FeSO_4_·7H_2_O | 0.16 |
| diethylenetriaminepentaacetic acid | 0.22 |

**Table S3** Modified Sonneveld's recipe for the hydroponic solution (tank B, Total Volume 2.5 L) [5]

| **Salt** | **Weight [g]** |
| --- | --- |
| KH_2_PO_4_ | 34.10 |
| MgSO_4_·7H_2_O | 61.60 |
| MnSO_4_·H_2_O | 0.19 |
| H_3_BO_3_ | 0.23 |
| Na_2_MoO_4_·2H_2_O | 0.02 |
| ZnSO_4_·7H_2_O | 0.14 |
| CuSO_4_·5H_2_O | 0.02 |

**Table S4** Sampling location and physico-chemical properties of soil

| **Sampling location** | |
| --- | --- |
| State | Czech Republic |
| Region | The Vysočina region |
| Town | Jemnice |
| Sampling depth [cm] | 0-25 |
| **Physico-chemical properties of soil** | |
| Soil texture | Sandy Loam |
| Soil type | Fluvisol |
| Sand [%] | 52.05 |
| Silt [%] | 32.65 |
| Clay [%] | 15.30 |
| pH _(CaCl2)_ [-] | 6.47 |
| pH _(H2O)_ [-] | 7.37 |
| EC [mS∙cm^-1^] | 0.287 |
| Maximal water holding capacity [%] | 44 |
| Exchangable Mg^2+^ [mg∙kg-1] | 57.1 |
| Exchangable Ca^2+^ [mg∙kg^-1^] | 388.2 |
| Exchangable K^+^ [mg∙kg^-1^] | 135.8 |
| Exchangable Na^+^ [mg∙kg^-1^] | 45.0 |
| Exchangable NH_4_^+^ [mg∙kg^-1^] | 6.8 |
| Exchangable NO_3_^-^ [mg∙kg^-1^] | 189.0 |
| Exchangable PO_4_^3-^ [mg∙kg^-1^] | 194.2 |
| Total phosphorus – water extract [mg∙kg^-1^] | 46.7 |
| Total Nitrogen – water extract [%] | 0.128 |
| Total Carbon [%] | 2.50 |
| Inorganic Carbon [%] | 0.27 |
| Organic carbon [%] | 2.23 |
| Organic matter [%] | 3.83 |
| Ca - Aqua regia [mg∙kg^-1^] | 388.2 |
| K - Aqua regia [mg∙kg^-1^] | 1,594 |
| Mg - Aqua regia [mg∙kg^-1^] | 7,158 |
| Na - Aqua regia [mg∙kg^-1^] | 87.1 |
| NH_4_^+^ - Aqua regia [mg∙kg^-1^] | 89.6 |
| Total phosphorus - Aqua regia [mg∙kg^-1^] | 2,822 |
| PO_4_^3-^ - Aqua regia [mg∙kg^-1^] | 1,973 |


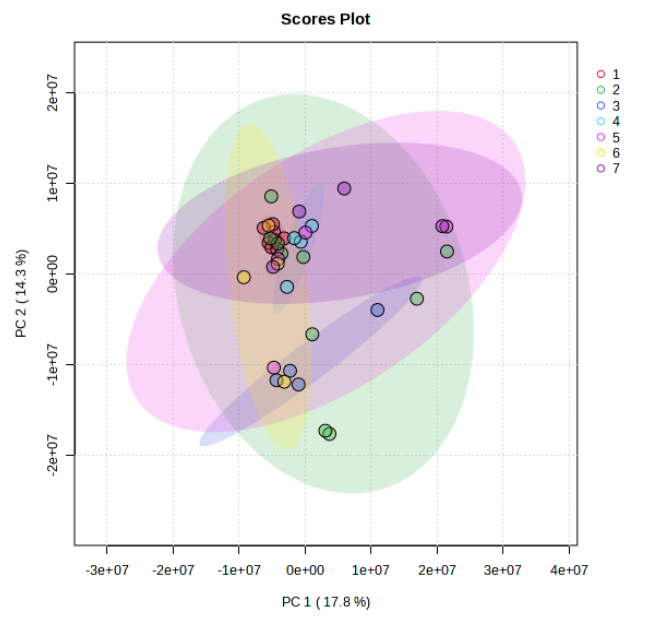


**BLANK**

**21 DAYS**

**7 DAYS**

**3 DAYS**

**1 DAY**

**QC**

**14 DAYS**

**QC**

**BLANK**

**1 DAY**

**3 DAYS**

**7 DAYS**

**14 DAYS**

**21 DAYS**

**Fig. S1** PCA of earthworm samples measured in DDA+ mode using LC-qTOF (QC – red; BLANK – green; 1 DAY – dark blue; 3 DAYS – light blue; 7 DAYS - pink; 14 DAYS yellow; 21 DAYS – purple)
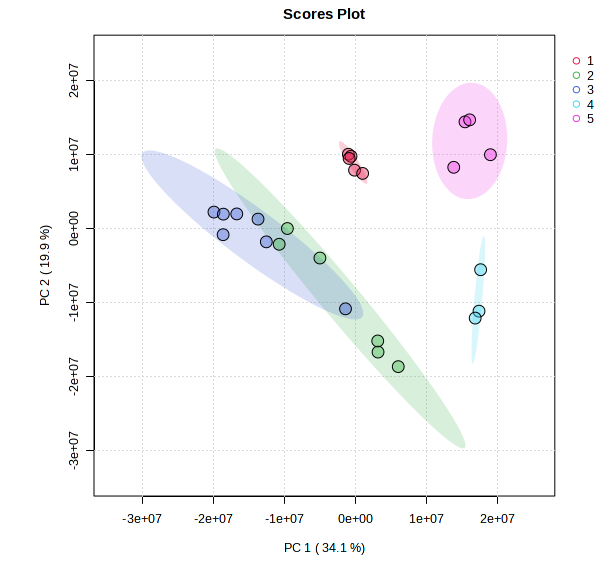


**QC**

**SAMPLE 28D**

**SAMPLE 21D**

**BLANK 28D**

**BLANK 21D**

**QC**

**BLANK 21D**

**BLANK 28D**

**SAMPLE 21D**

**SAMPLE 28 D**

**Fig. S2** PCA of soil grown lettuce - roots samples measured in DDA+ mode using LC-qTOF (QC – red; BLANK 21D – green; SAMPLE 21D – light blue; BLANK 28D – dark blue; SAMPLE 28D – pink)
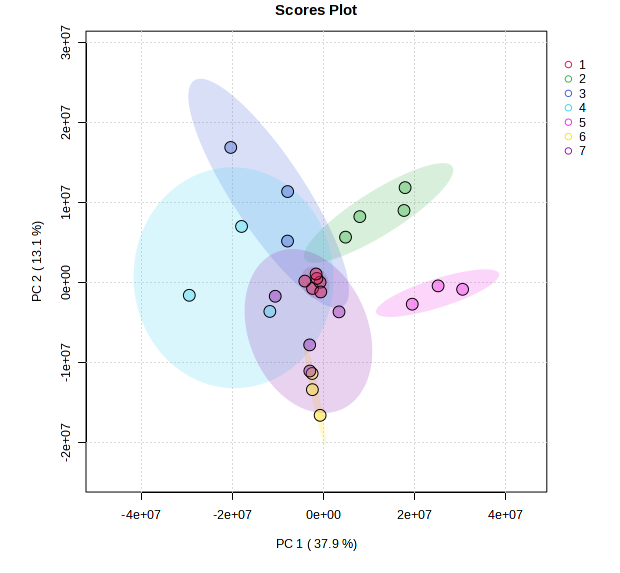


**SAMPLE 28D**

**SAMPLE 21D**

**SAMPLE 14D**

**BLANK 14D**

**BLANK 21D**

**BLANK 28D**

**QC**

**Fig. S3** PCA of soil grown lettuce - leaves samples (14D samples consists of whole lettuce sampling) measured in DDA+ mode using LC-qTOF (QC – red; BLANK 14D – green; SAMPLE 14D – pink; BLANK 21D – dark blue; SAMPLE 21D – yellow; BLANK 28D – light blue; SAMPLE 28D – purple)

**Table S5** The quantity of significant features in volcano plot (p<0.05, FC > 3) specific to individual sample types across various MS/MS modes

| **Sample Type** | **DIA+** | **DIA-** | **DDA+** | **DDA-** |
| --- | --- | --- | --- | --- |
| Earthworms | 545 of 4,283 | 146 of 1,507 | 1,293 of 8,177 | 218 of 2,021 |
| Hydroponics lettuce roots | 903 of 6,043 | 410 of 2,770 | 3,539 of 11,748 | 934 of 3,034 |
| Hydroponics lettuce leaves | 169 of 3,222 | 142 of 2,457 | 516 of 12,222 | 356 of 5,958 |
| Soil lettuce roots | 1,019 of 12,691 | 718 of 6,442 | 2,067 of 22,042 | 789 of 7,975 |
| Soil lettuce leaves | 621 of 11,556 | 492 of 4,282 | 1,239 of 20,231 | 449 of 5,294 |

**Table S6** Overview of identified metabolites in earthworms and lettuce samples by LC-qTOF

| Compound Name | Metabolic Reaction | RT  [min] | Chemical Formula | Monoisotopic  Mw [-] | **Positive Ionisation Mode** | | **Negative Ionisation Mode** | | **Confidence level** |
| --- | --- | --- | --- | --- | --- | --- | --- | --- | --- |
|  |  |  |  |  | Precursor Ion - m/z | Product Ions - m/z | Precursor  Ion - m/z | Product Ions - m/z |  |
| Atenolol | - | 2.94 | C_14_H_22_N_2_O_3_ | 266.163043 | 267.1699 | 250.1434; 225.1236; 208.966; 190.0865; 178.0858; 145.0646; 134.0637; 116.1062; 98.096; 74.0592; 72.0799 | - | - | Level 1 |
| Atenolol-LS1 | 1x Oxidative deamination | 4.22 | C_14_H_21_NO_4_ | 267.1471 | 268.1553 | 250.1471; 226.1073; 191.0704; 179.0679; 165.0557; 145.0647; 137.0588; 116.1061; 107.0488; 98.0956; 74.0593; 72.0806 | - | - | Level 2 |
| Enrofloxacin | - | 5.23 | C_19_H_22_FN_3_O_3_ | 359.16452 | 360.1715 | 342.1616; 316.1807; 245.1082; 231.0959 | - | - | Level 1 |
| Enrofloxacin-R63 | 1x Glucosidation | 3.80 | C_25_H_32_FN_3_O_8_ | 521.217345 | 522.2222 | 360.1689; 342.1599 | - | - | Level 3 |
| Enrofloxacin-R13 | 1x Methylation | 5.19 | C_20_H_24_FN_3_O_3_ | 373.18017 | 374.1874 | 360.1672; 342.1617; 316.1844; 245.1082; 231.0927 | - | - | Level 3 |
| Ciprofloxacin | 1x de-Alkylation | 4.97 | C_17_H_18_FN_3_O_3_ | 331.13322 | 332.1417 | 314.1317; 288.1487; 245.1055; 231.0564 | - | - | Level 1 |
| Enrofloxacin-R2 | 1x de-Carboxylation | 5.18 | C_18_H_22_FN_3_O | 315.17469 | 316.1810 | 245.1096 | - | - | Level 3 |
| Enrofloxacin-M242 | 1x Ring Cleavage | 4.79 | C_16_H_18_FN_3_O_3_ | 319.13322 | 320.1405 | 302.1322 | - | - | Level 3 |
| Erythromycin | - | 8.53 | C_37_H_67_NO_13_ | 733.461244 | 734.4684 | 716.4612; 576.3774; 558.3588; 540.3536; 158.1171 | - | - | Level 1 |
| Erythromycin-M1 | 1x de-Hydrogenation | 9.20 | C_37_H_65_NO_13_ | 731.445594 | 732.4558 | 574.3547; 158.1179 | - | - | Level 2 |
| Erythromycin-M46 | 1x de-Glycosylation | 7.87 | C_29_H_51_NO_10_ | 573.351299 | 574.3599 | 556.3390; 538.3452; 158.1172; 113.0594 | - | - | Level 2 |
| Erythromycin-M361 | 1x Hydrolysis;  1x de-Glycosylation | 6.09 | C_29_H_53_NO_11_ | 591.361864 | 592.3709 | 574.3640; 158.1181 | - | - | Level 2 |
| Ketoprofen | - | 9.54 | C_16_H_14_O_3_ | 254.094295 | 255.1021 | 209.0961; 177.0557; 105.0333; 77.07384 | 253.0856 | 209.0972 | Level 1 |
| Ketoprofen-R13 | 1x Methylation | 8.57 | C_17_H_16_O_3_ | 268.10994 | 269.1177 | 209.987; 105.0328; 77.037 | 267.1028 | 209.0983 | Level 3 |
| Ketoprofen-R23 | 1x Acetylation | 8.56 | C_18_H_16_O_4_ | 296.104855 | 297.1123 | 209.0961; 105.0335; 77.0382 | 295.0967 | 209.0963; 181.065 | Level 3 |
| Ketoprofen-R63 | 1x Glucosidation | 8.56 | C_22_H_24_O_8_ | 416.147115 | - | - | 415.1390 | 253.0849; 209.0966 | Level 3 |
| Ketoprofen-R81 | 1x Acetylation,  1x Glucosidation | 8.67 | C_24_H_26_O_9_ | 458.15768 | - | - | 457.1505 | 253.0866; 209.097 | Level 3 |
| Ketoprofen-R87 | 1x Malonic acid,  1x Glucosidation | 8.68 | C_25_H_26_O_11_ | 502.14751 | 503.1554 | 255.1025; 209.0962; 105.0328 | - | - | Level 3 |
| Ketoprofen-R93 | 2x Glucosidation | 8.55 | C_28_H_34_O_13_ | 578.19994 | - | - | 577.1926 | 253.0897; 209.0974 | Level 3 |
| Ketoprofen-R96 | 1x Hydroxylation,  2x Glucosidation | 8.4 | C_28_H_34_O_14_ | 594.194855 | - | - | 593.1868 | 253.0872; 209.097 | Level 3 |
| Ketoprofen-R111 | 1x Hydroxylation,  4x Glucosidation | 8.59 | C_40_H_54_O_24_ | 918.300505 | - | - | 917.2899 | 253.083; 209.0972 | Level 3 |
| Ketoprofen-LS24 | 1x de-Carboxylation | 8.22 | C_15_H_14_O | 210.104465 | - | - | 209.0970 | 181.0657 | Level 2 |
| Ketoprofen-LS25 | 1x de-Carboxylation,  1x Oxidation | 8.69 | C_15_H_12_O_2_ | 224.08373 | - | - | 223.0768 | 209.096 | Level 2 |
| Ketoprofen-M648 | 1x de-Carboxylation  1x de-Hydrogenation | 8.66 | C_15_H_14_O_3_ | 242.094295 | - | - | 241.0880 | 197.0607 | Level 3 |
| Ketoprofen-M835 | 1x de-Carboxylation  2x Hydroxylation | 9.55 | C_15_H_12_O | 208.088815 | 209.0956 | 131.0494; 105.0316; 77.0373 | - | - | Level 3 |
| Sulfamethoxazole | - | 5.15 | C_10_H_11_N_3_O_3_S | 253.052114 | 254.0595 | 156.0105; 108.0438; 92.0503 | 252.0451 | 156.0124; 92.0508 | Level 1 |
| Sulfamethoxazole-LS1 | 1x Acetylation | 6.25 | C_12_H_13_N_3_O_4_S | 295.062679 | 296.0712 | 198.0222; 156.0591; 134.0591; 108.0438 | 294.0594 | 134.0599 | Level 2 |
| Sulfamethoxazol-R63 | 1x Glucosidation | 4.45 | C_16_H_21_N_3_O_8_S | 415.104939 | 416.1125 | 318.0634; 254.0593; 156.0108; 108.0433; 92.0505 | 414.09753 | 252.2446; 156.0104; 134.0613; 92.0505 | Level 2 |
| Sulfamethoxazole-LS6 | 1x Pterin Conjugation | 6.09 | C_17_H_16_N_8_O_4_S | 428.101524 | 429.1079 | 331.0615; 267.0996; 176.0564 | - | - | Level 2 |
| Tetracycline | - | 4.30 | C_22_H_24_N_2_O_8_ | 444.153268 | 445.1617 | 427.1485; 428.1434; 410.1243; 392.1115 | 443.1459 | 426.1174; 358.1278; 273.076; 271.0651 | Level 1 |
| Tetracycline-LS2 | 1x de-Amination;  1x de-Hydratation | 4.27 | C_22_H_19_NO_7_ | 409.116154 | 410.1233 | 392.1122 | - | - | Level 2 |
| Tetracycline-LS3 | 1x de-Hydratation | 4.27 | C_22_H_22_N_2_O_7_ | 426.142703 | 427.1503 | 410.1237; 392.1124 | - | - | Level 2 |

**Table S7** Overview of the method of prediction for identified metabolites and their SMILES codes

| **Compound Name** | **SMILES** | **Metabolic Reaction** | **Method of Prediction** | | |
| --- | --- | --- | --- | --- | --- |
|  |  |  | **Software Prediction** | **Literature Search** | **Common Reaction Pathways** |
| Atenolol | CC(C)NCC(COC1=CC=C(C=C1)CC(=O)N)O | - | - | - | - |
| Atenolol acid | CC(C)NCC(O)COc1ccc(cc1)CC(=O)O | 1x Oxidative deamination | Atenolol-M78 | Atenolol-LS1 | Atenolol-R128 |
| Enrofloxacin | CCN1CCN(CC1)C2=C(C=C3C(=C2)N(C=C(C3=O)C(=O)O)C4CC4)F | - | - | - | - |
| Enrofloxacin-R63 | O=C(OC1OC(CO)C(O)C(O)C1O)C5=CN(C2CC2)c4cc(N3CCN(CC)CC3)c(F)cc4C5=O | 1x Glucosidation | x | x | Enrofloxacin-R63 |
| Enrofloxacin-R13 | - | 1x Methylation | x | x | Enrofloxacin-R13 |
| Ciprofloxacin | C1CC1N2C=C(C(=O)C3=CC(=C(C=C32)N4CCNCC4)F)C(=O)O | 1x de-Alkylation | Enrofloxacin-M3 | Ciprofloxacin | x |
| Enrofloxacin-R2 | CCN1CCN(CC1)c2cc3N(C=CC(=O)c3cc2F)C4CC4 | 1x de-Carboxylation | x | x | Enrofloxacin-R2 |
| Enrofloxacin-M242 | CCN1CCN(CC1)c2cc3NC=C(C(=O)O)C(=O)c3cc2F | 1x Ring Cleavage | Enrofloxacin-M242 | x | x |
| Erythromycin | CCC1C(C(C(C(=O)C(CC(C(C(C(C(C(=O)O1)C)OC2CC(C(C(O2)C)O)(C)OC)C)OC3C(C(CC(O3)C)N(C)C)O)(C)O)C)C)O)(C)O | - | - | - | - |
| Erythromycin-M1 | CCC1C(C(C(C(=O)C(CC(C(C(C(C(C(=O)O1)C)OC2CC(C(C(O2)C)O)(C)OC)C)OC3C(C(CC(O3)C)N(C)C)O)(C)O)C)C)=O)(C)O | 1x de-Hydrogenation | Erythromycin-M1 | x | Erythromycin-R130 |
| Erythromycin-M46 | CCC1C(C(C(C(=O)C(CC(C(C(C(C(C(=O)O1)C)=O)C)OC2C(C(CC(O2)C)N(C)C)O)(C)O)C)C)O)(C)O | 1x de-Glycosylation | Erythromycin-M46 | x | x |
| Erythromycin-M361 | CCC(O)C(C)(O)C(O)C(C)C(=O)C(C)CC(C)(O)C(OC1OC(C)CC(C1O)N(C)C)C(C)C(=O)C(C)C(=O)O | 1x Hydrolysis; 1x de-Glycosylation | Erythromycin-M361 | x | x |
| Ketoprofen | CC(C1=CC(=CC=C1)C(=O)C2=CC=CC=C2)C(=O)O | - | - | - | - |
| Ketoprofen-R13 | - | 1x Methylation | x | x | Ketoprofen-R13 |
| Ketoprofen-R23 | - | 1x Acetylation | x | x | Ketoprofen-R23 |
| Ketoprofen-R63 | O=C(OC1OC(CO)C(O)C(O)C1O)C(C)c2cccc(c2)C(=O)c3ccccc3 | 1x Glucosidation | x | Ketoprofen-LS14 | Ketoprofen-R63 |
| Ketoprofen-R81 | - | 1x Acetylation, 1x Glucosidation | x | x | Ketoprofen-R81 |
| Ketoprofen-R87 | O=C(OC1OC(COC(=O)CC(=O)O)C(O)C(O)C1O)C(C)c2cccc(c2)C(=O)c3ccccc3 | 1x Malonic acid, 1x Glucosidation | x | Ketoprofen-LS18 | Ketoprofen-R87 |
| Ketoprofen-R93 | O=C(OC2OC(COC1OC(CO)C(O)C(O)C1O)C(O)C(O)C2O)C(C)c3cccc(c3)C(=O)c4ccccc4 | 2x Glucosidation | x | Ketoprofen-LS10 | Ketoprofen-R93 |
| Ketoprofen-R96 | - | 1x Hydroxylation, 2x Glucosidation | x | x | Ketoprofen-R96 |
| Ketoprofen-R111 | - | 1x Hydroxylation, 4x Glucosidation | x | x | Ketoprofen-R111 |
| Ketoprofen-LS24 | CCc1cccc(c1)C(=O)c2ccccc2 | 1x de-Carboxylation | Ketoprofen-M35 | Ketoprofen-LS24 | Ketoprofen-R2 |
| Ketoprofen-LS25 | O=C(C)c1cccc(c1)C(=O)c2ccccc2 | 1x de-Carboxylation,  1x Oxidation | Ketoprofen-M638 | Ketoprofen-LS25 | x |
| Ketoprofen-M648 | CC(C1=CC(=C(C=C1)O)C(=O)C2=CC=CC=C2)O | 1x de-Carboxylation  1x de-Hydrogenation | Ketoprofen-M648 | x | x |
| Ketoprofen-M835 | O=C(c1ccccc1)c2cccc(C=C)c2 | 1x de-Carboxylation  2x Hydroxylation | Ketoprofen-M835 | x | x |
| Sulfamethoxazole | CC1=CC(=NO1)NS(=O)(=O)C2=CC=C(C=C2)N | - | - | - | - |
| Sulfamethoxazole-LS1 | Cc2cc(NS(=O)(=O)c1ccc(NC(C)=O)cc1)no2 | 1x Acetylation | Sulfamethoxazole-M28 | Sulfamethoxazole-LS1  and Sulfamethoxazole-LS2 | Sulfamethoxazole-R23 |
| Sulfamethoxazol-R63 | Cc3cc(NS(=O)(=O)c2ccc(NC1OC(CO)C(O)C(O)C1O)cc2)no3 | 1x Glucosidation | x | Sulfamethoxazole-LS4 | Sulfamethoxazole-R63 |
| Sulfamethoxazole-LS6 | Cc4cc(NS(=O)(=O)c3ccc(NCc1cnc2C(=O)NC(N)=Nc2n1)cc3)no4 | 1x Pterin Conjugation | x | Sulfamethoxazole-LS6 | x |
| Tetracycline | CC1(C2CC3C(C(=O)C(=C(C3(C(=O)C2=C(C4=C1C=CC=C4O)O)O)O)C(=O)N)N(C)C)O | - | - | - | - |
| Tetracycline-LS2 | CN(C)[C@@H]2C(=O)/C(=C=O)C(=O)[C@@]1(O)C(=O)c3c(C[C@H]12)c(C)c4cccc(O)c4c3O | 1x de-Amination; 1x de-Hydratation | x | Tetracycline-LS2 | x |
| Tetracycline-LS3 | NC(=O)C3=C(O)[C@@H](N(C)C)[C@@H]4Cc2c(c(O)c1c(O)cccc1c2C)C(=O)[C@]4(O)C3=O | 1x de-Hydratation | x | Tetracycline-LS3 | x |


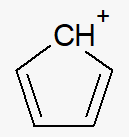

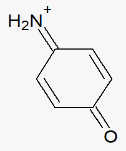

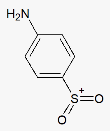

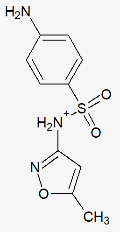

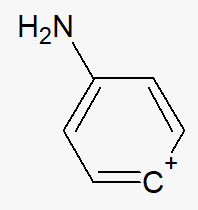

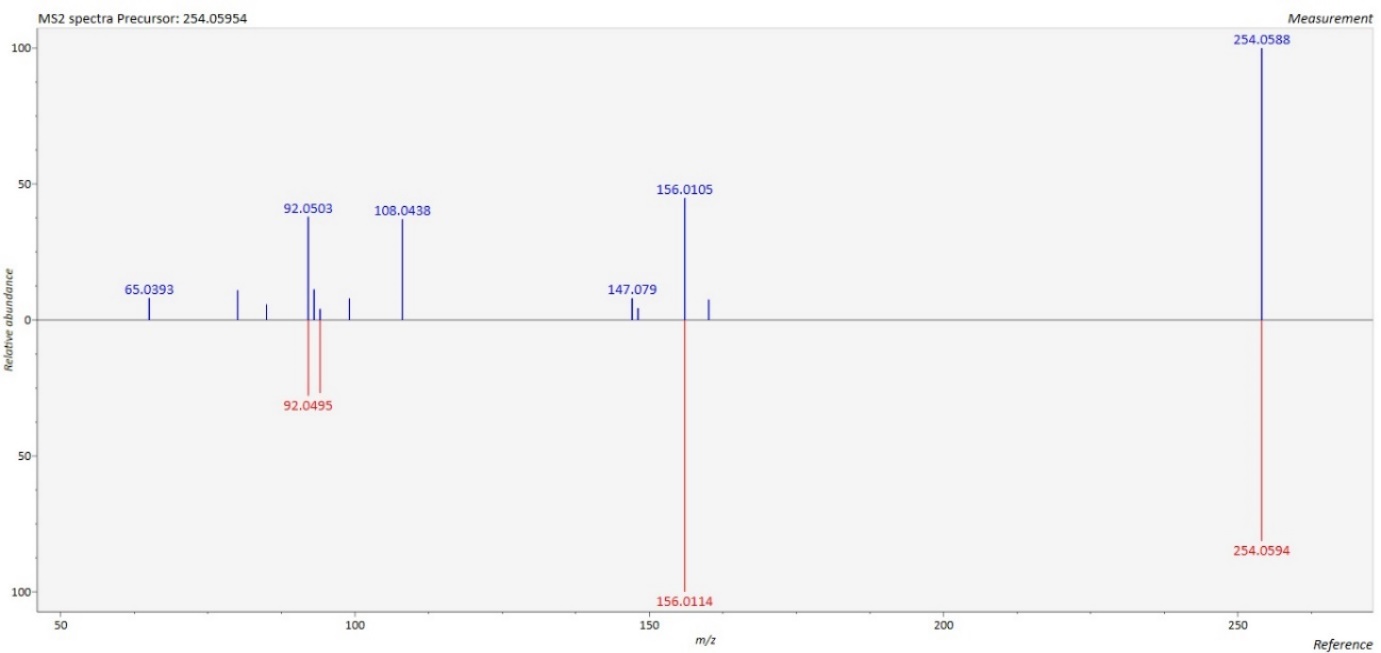


**Fig. S4** MS/MS spectra of Sulfamethoxazole in ESI+, Library match with in-silico predicted MS/MS spectra


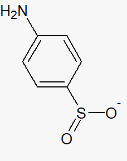

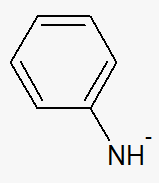

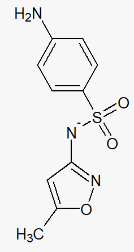

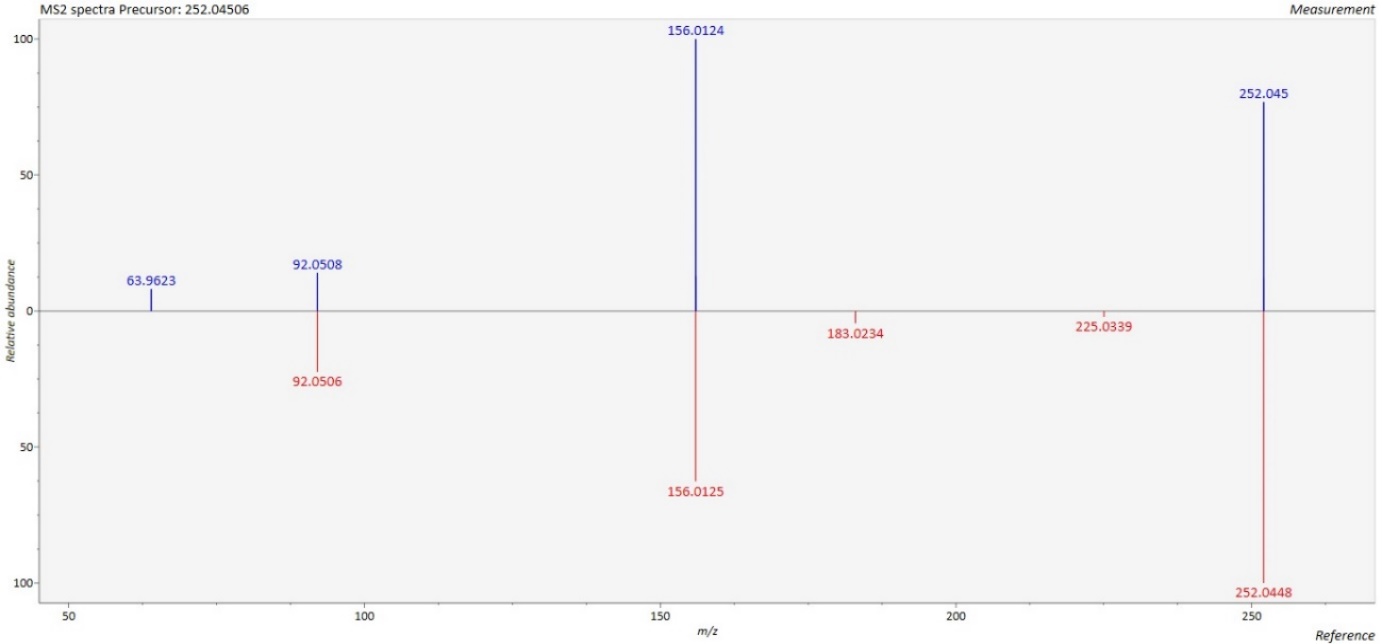


**Fig. S5** MS/MS spectra of Sulfamethoxazole in ESI-, Library match with in-silico predicted MS/MS spectra


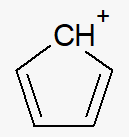

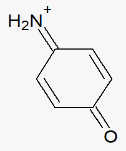

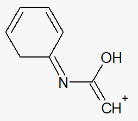

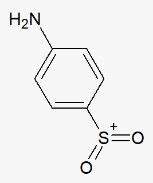

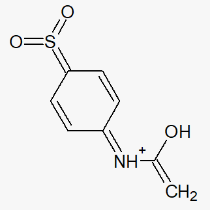

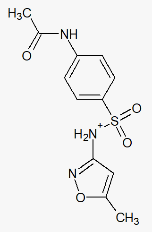

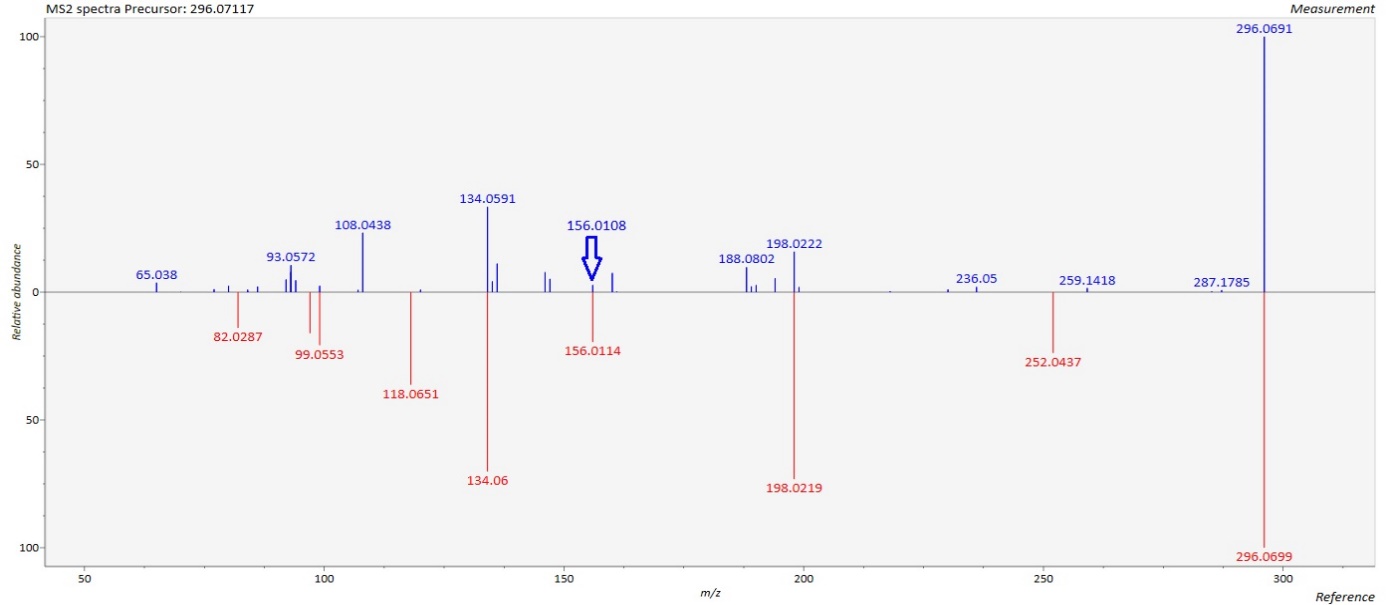


**Fig. S6** MS/MS spectra of Sulfamethoxazole-LS1 in ESI+, Library match with in-silico predicted MS/MS spectra


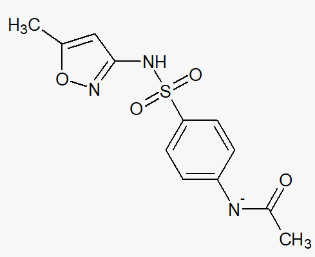

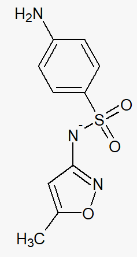

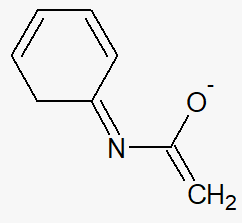

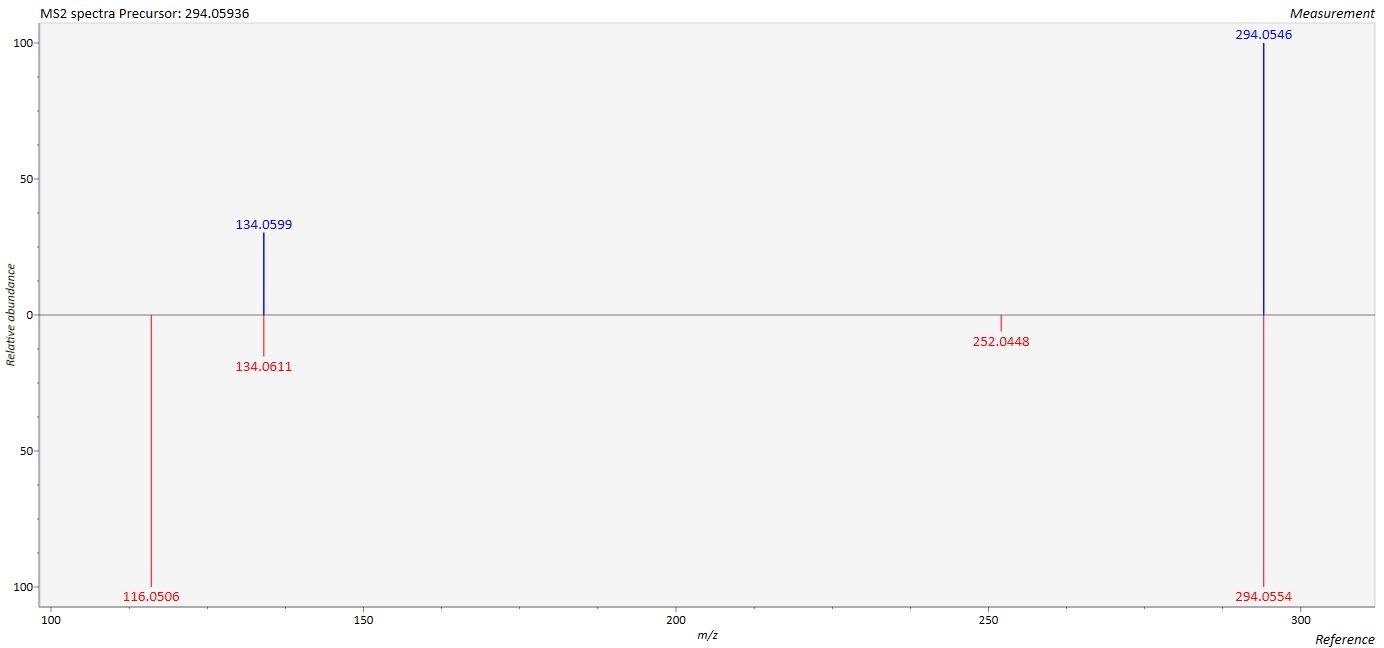


**Fig. S7** MS/MS spectra of Sulfamethoxazole-LS1 in ESI-, Library match with in-silico predicted MS/MS spectra


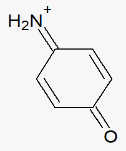

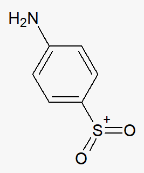

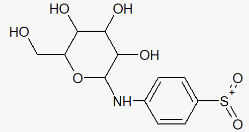

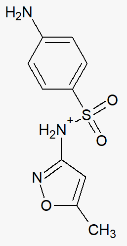

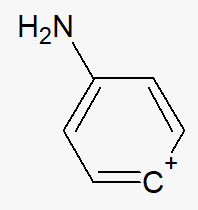

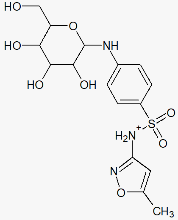

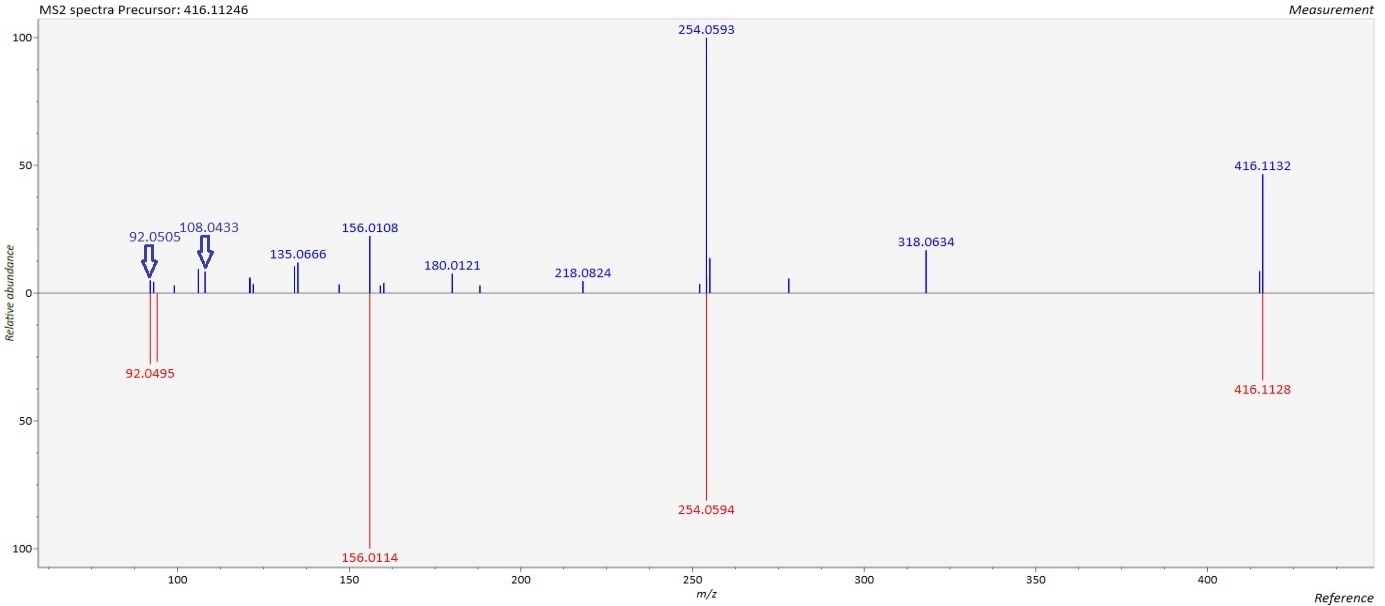


**Fig. S8** MS/MS spectra of Sulfamethoxazole-R63 in ESI+, Library match with in-silico predicted MS/MS spectra


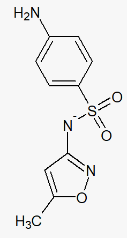

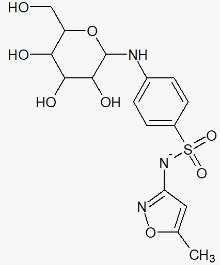

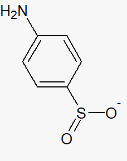

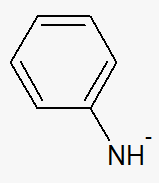

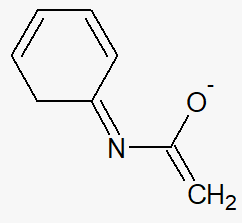

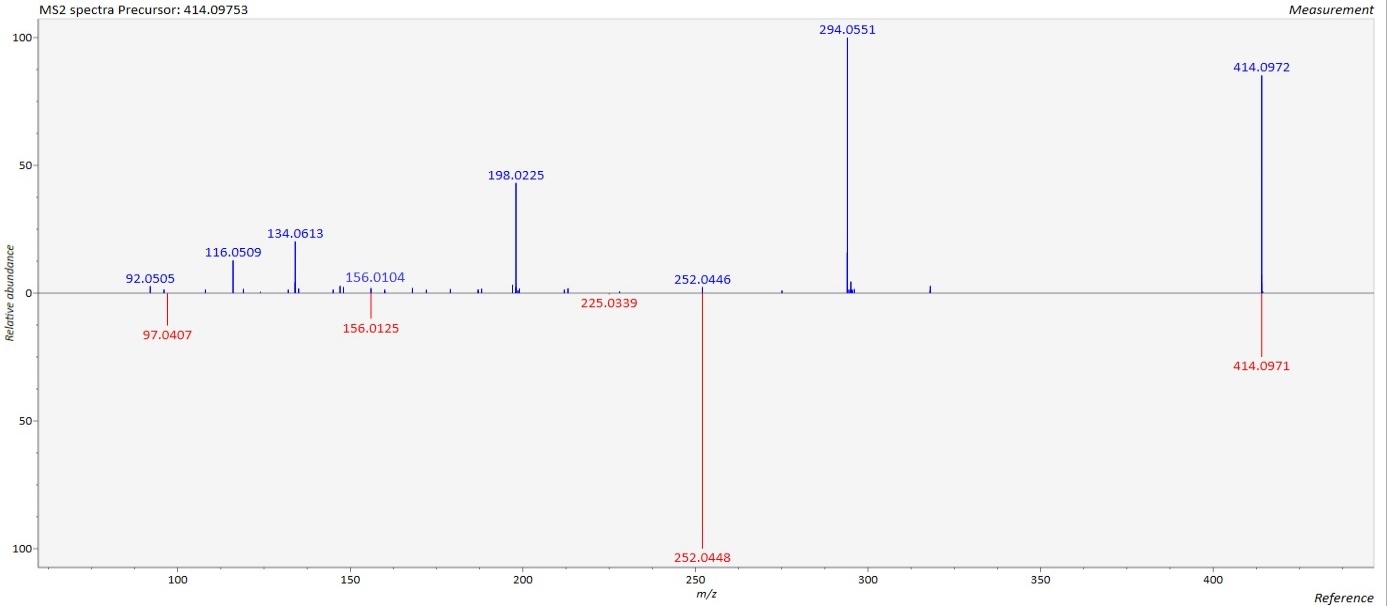


**Fig. S9** MS/MS spectra of Sulfamethoxazole-R63 in ESI-, Library match with in-silico predicted MS/MS spectra


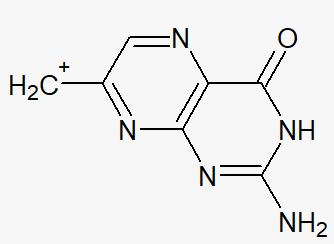

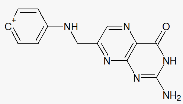

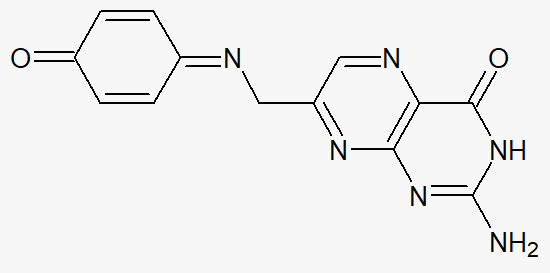

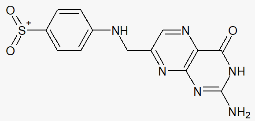

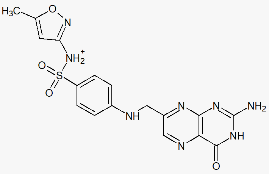

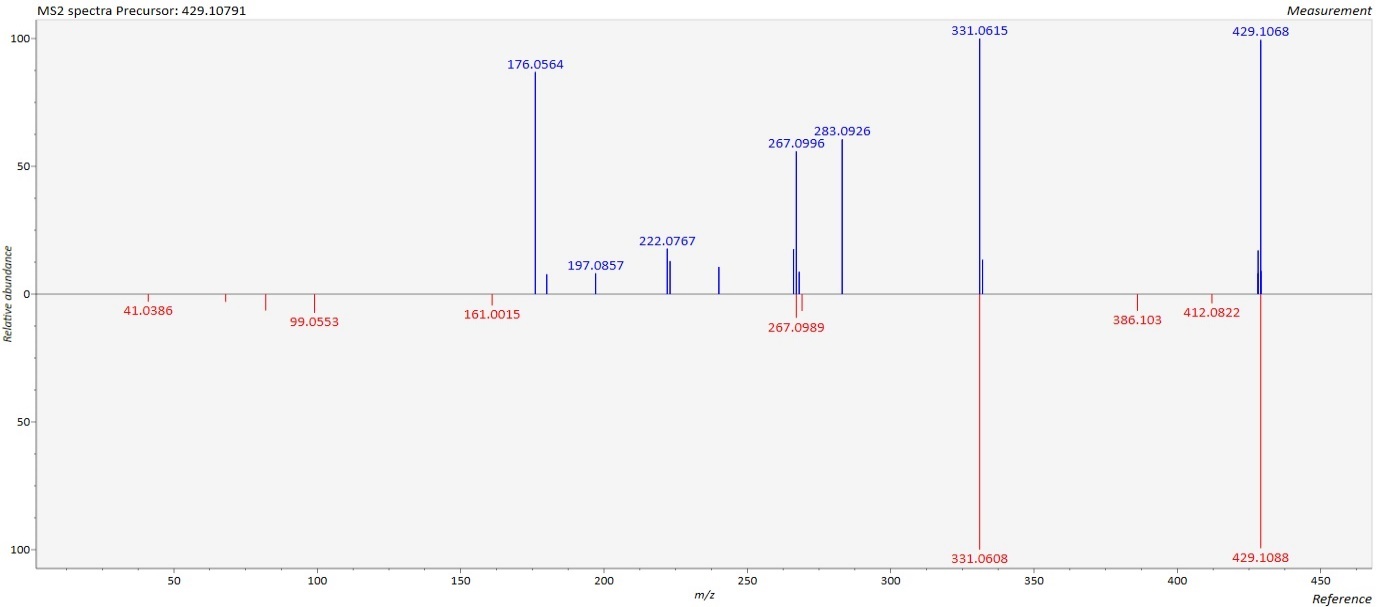


**Fig. S10** MS/MS spectra of Sulfamethoxazole-LS6 in ESI+, Library match with in-silico predicted MS/MS spectra


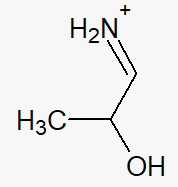

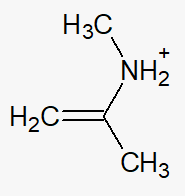

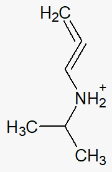

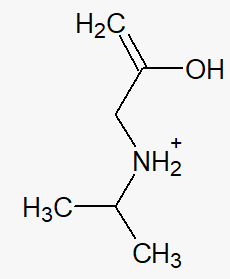

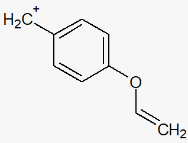

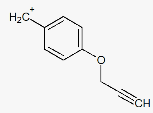

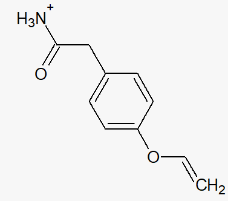

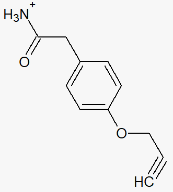

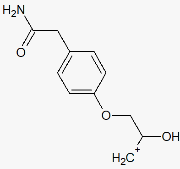

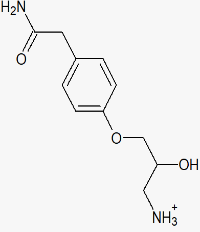

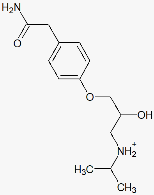

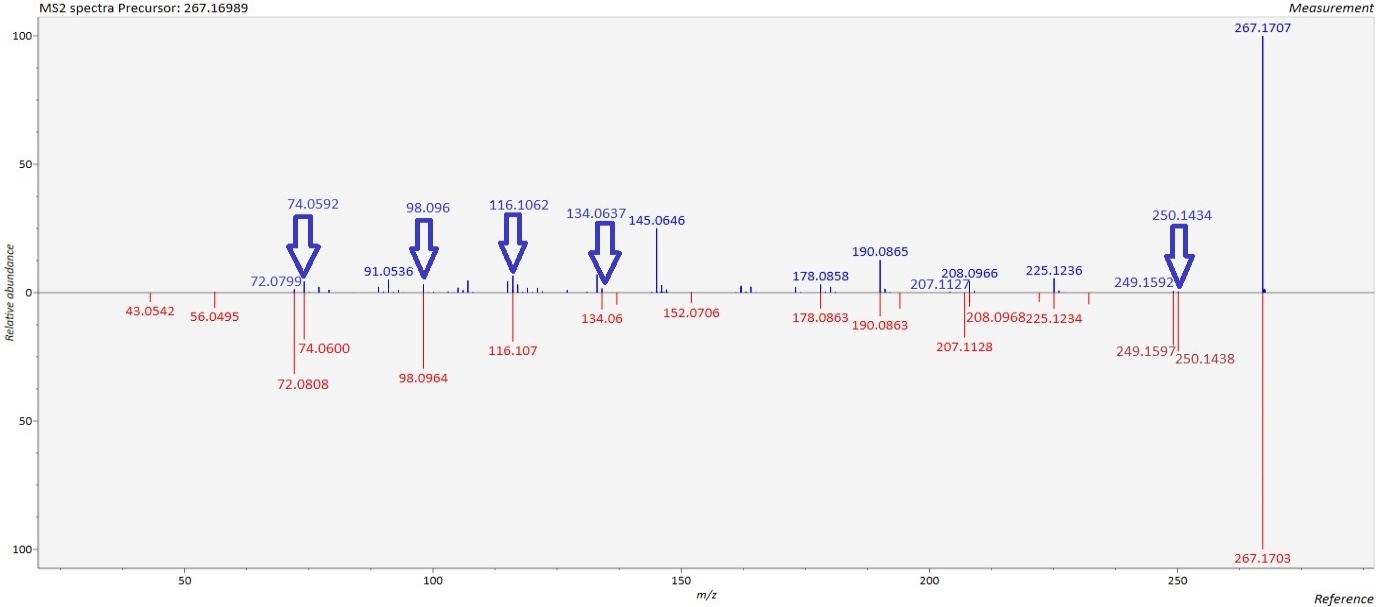


**Fig. S11** MS/MS spectra of Atenolol in ESI+, Library match with in-silico predicted MS/MS spectra


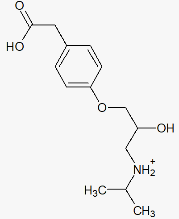

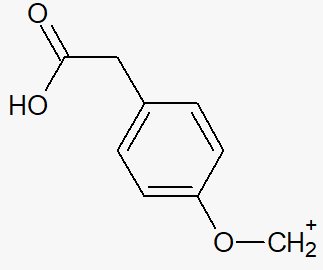

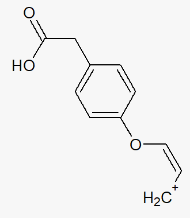

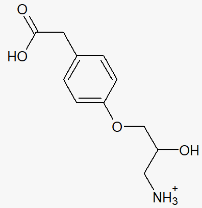

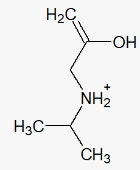

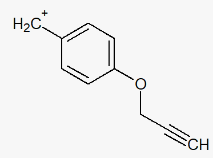

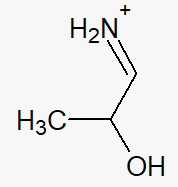

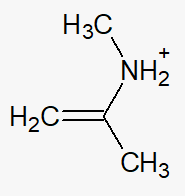

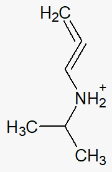

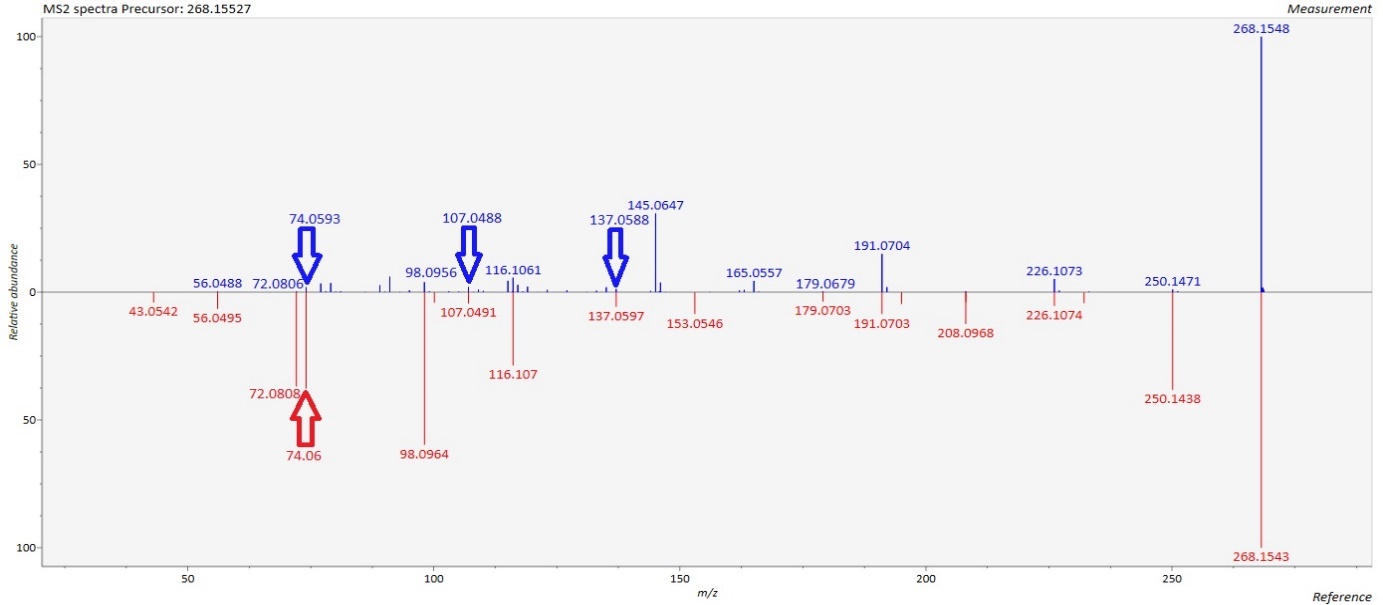


**Fig. S12** MS/MS spectra of Atenolol-LS1 in ESI+, Library match with in-silico predicted MS/MS spectra


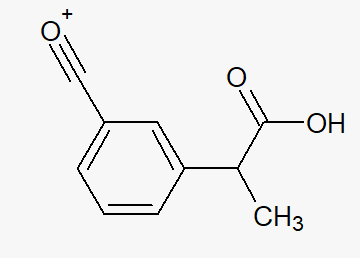

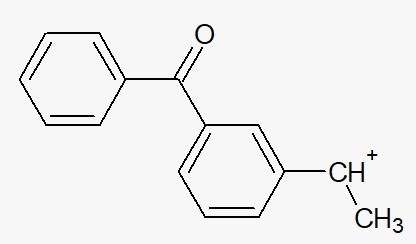

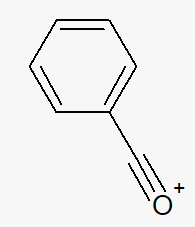

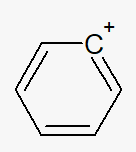

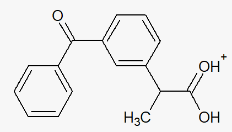

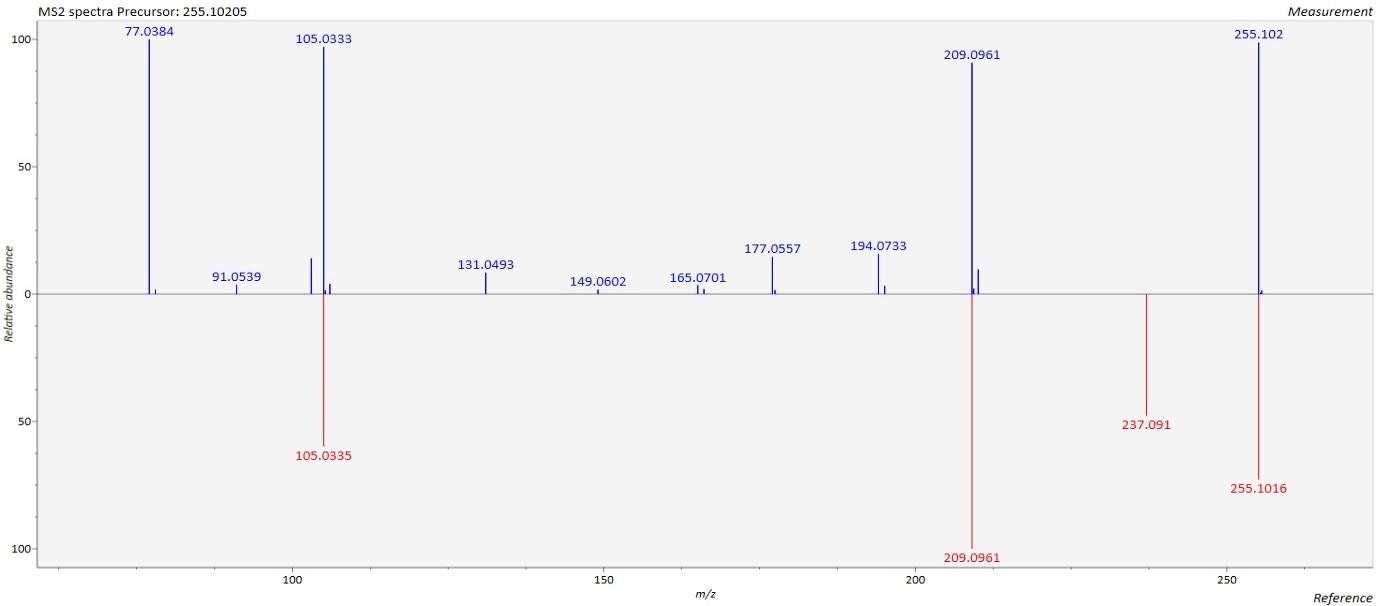
**Fig. S13** MS/MS spectra of Ketoprofen in ESI+, Library match with in-silico predicted MS/MS spectra


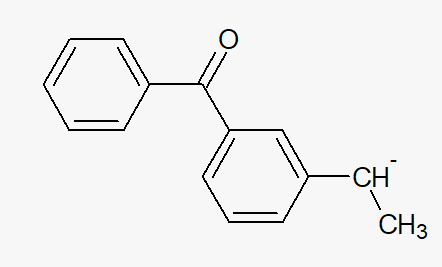

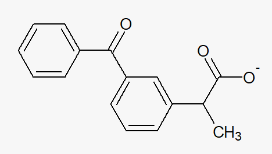

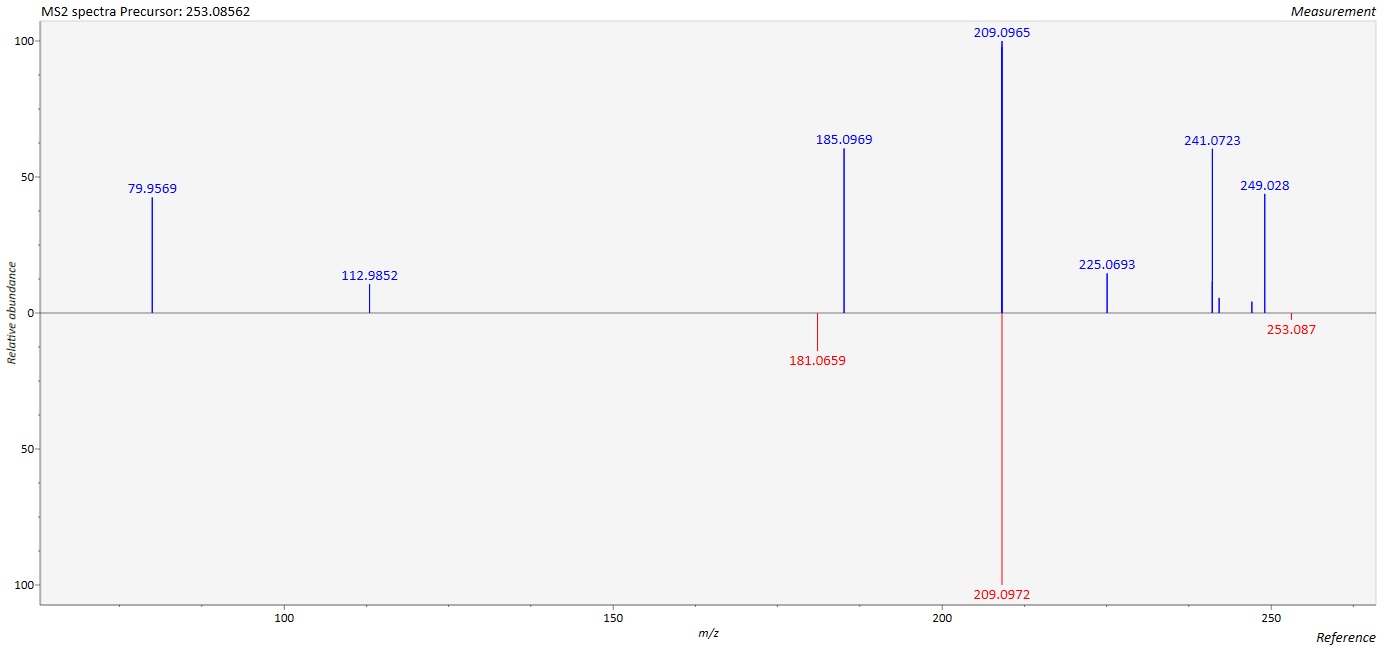


**Fig. S14** MS/MS spectra of Ketoprofen in ESI-, Library match with in-silico predicted MS/MS spectra


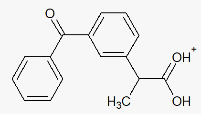

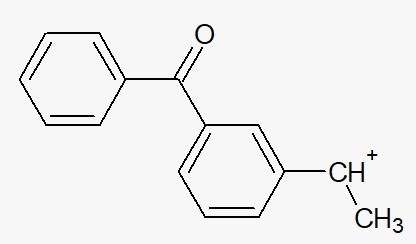

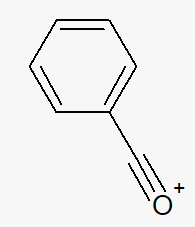

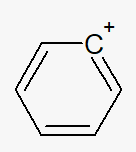

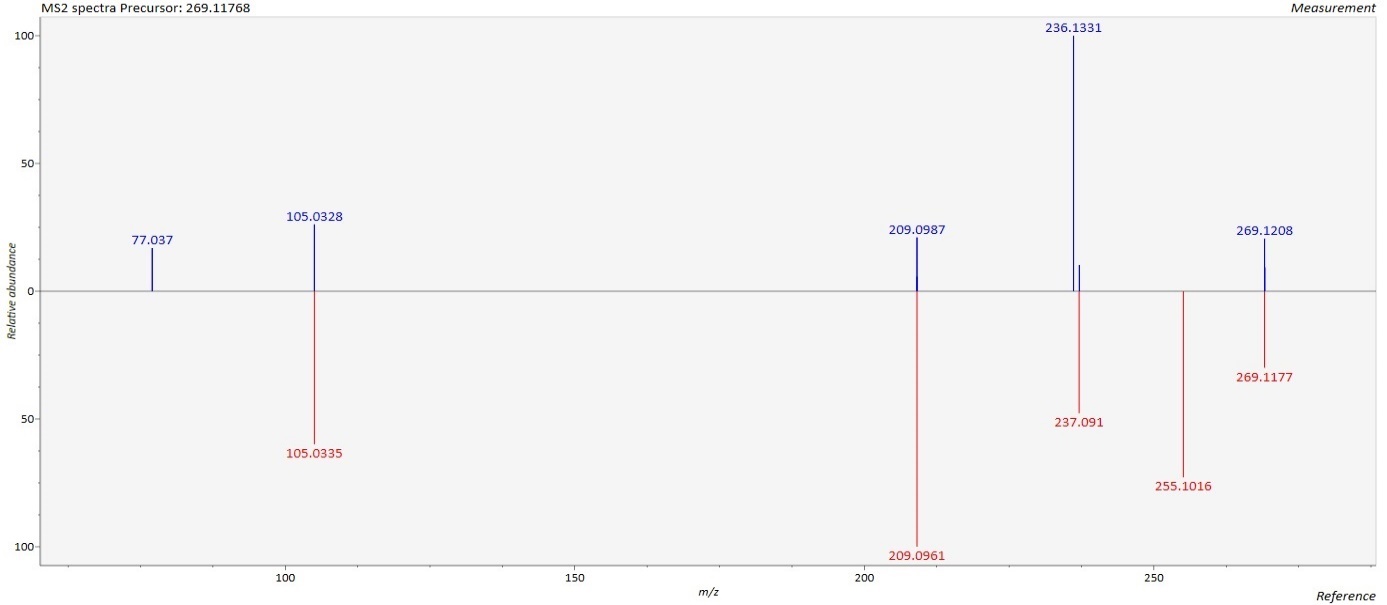


Methylated

Ketoprofen

[M+H]^+^

**Fig. S15** MS/MS spectra of Ketoprofen-R13 in ESI+, Library match with in-silico predicted MS/MS spectra


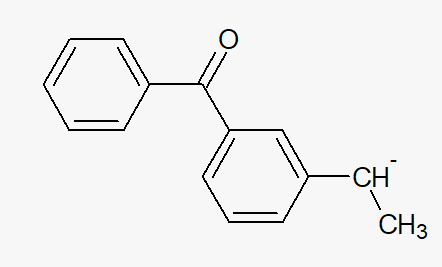

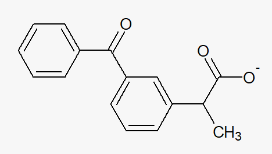

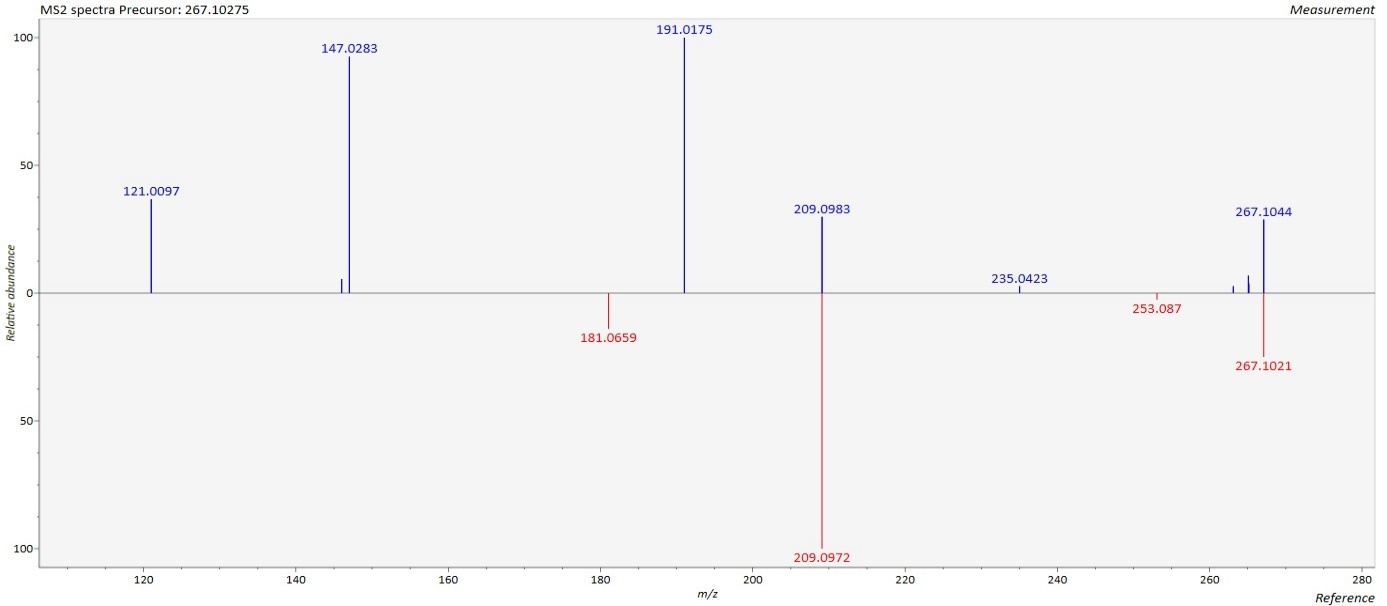


Methylated

Ketoprofen

[M-H]^-^

**Fig. S16** MS/MS spectra of Ketoprofen-R13 in ESI-, Library match with in-silico predicted MS/MS spectra


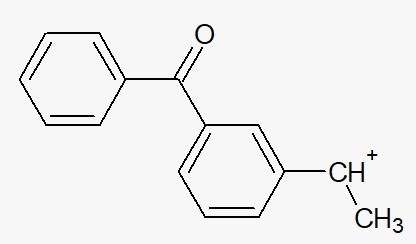

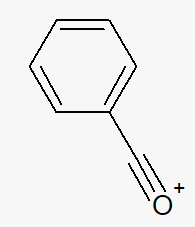

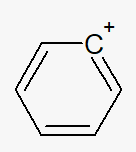

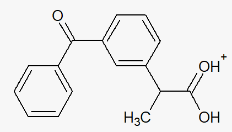

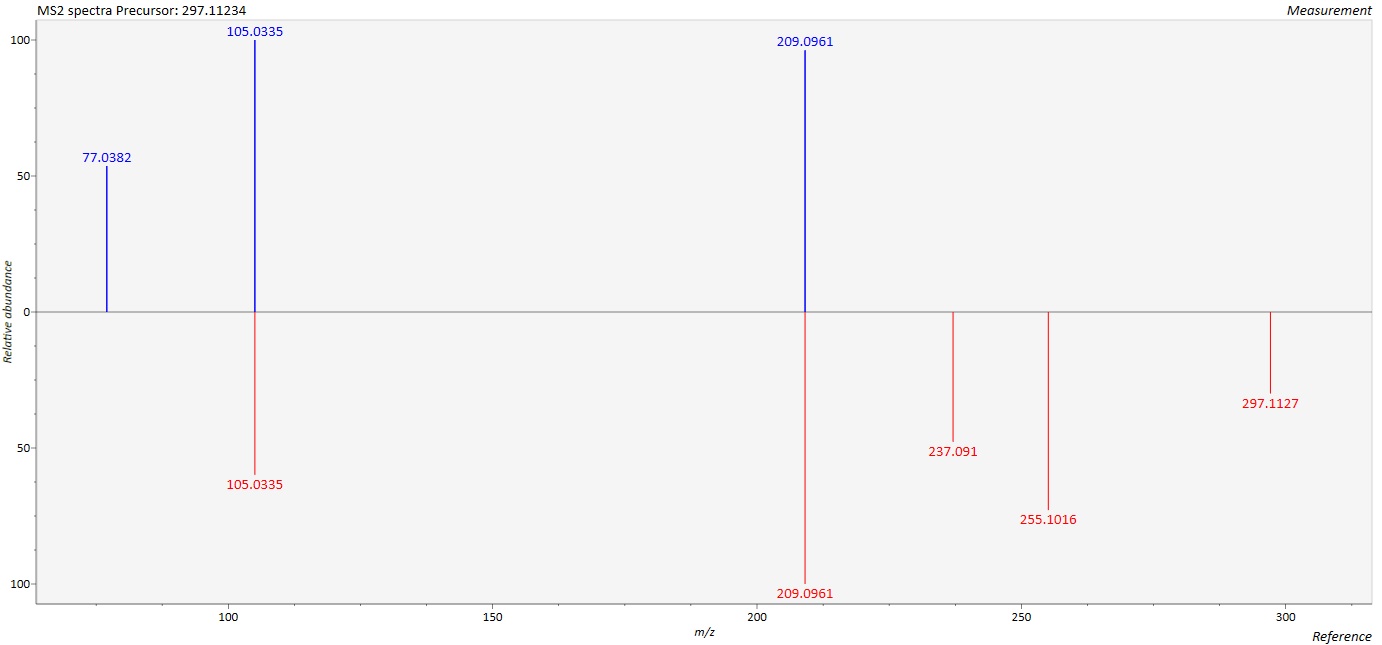


Acetylated

Ketoprofen

[M+H]^+^

**Fig. S17** MS/MS spectra of Ketoprofen-R23 in ESI+, Library match with in-silico predicted MS/MS spectra


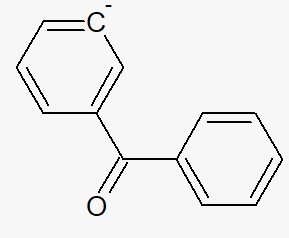

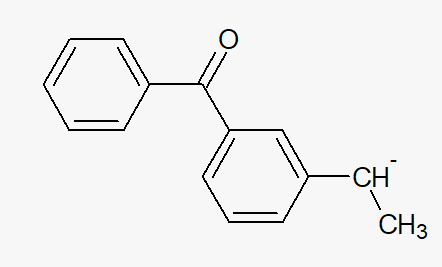

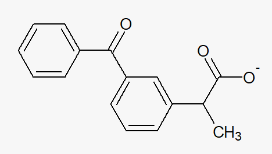

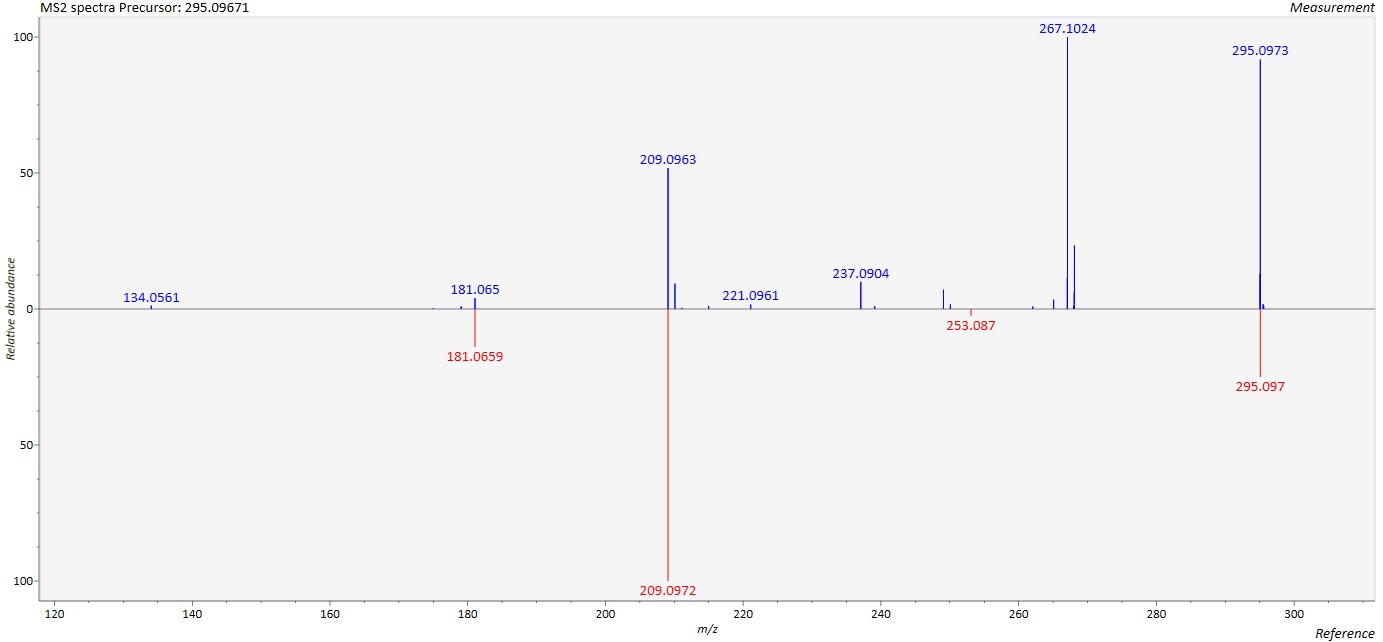


Acetylated

Ketoprofen

[M-H]^-^

**Fig. S18** MS/MS spectra of Ketoprofen-R23 in ESI-, Library match with in-silico predicted MS/MS spectra


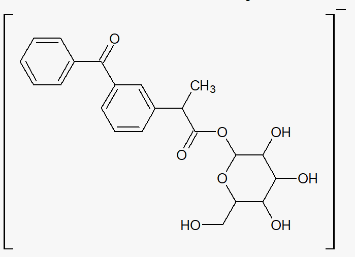

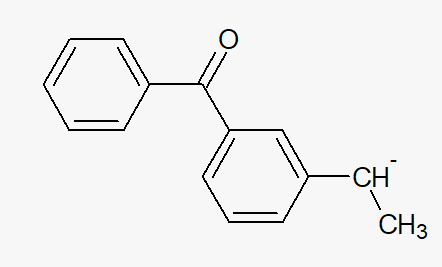

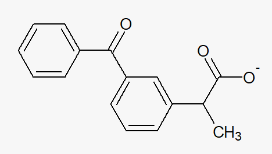

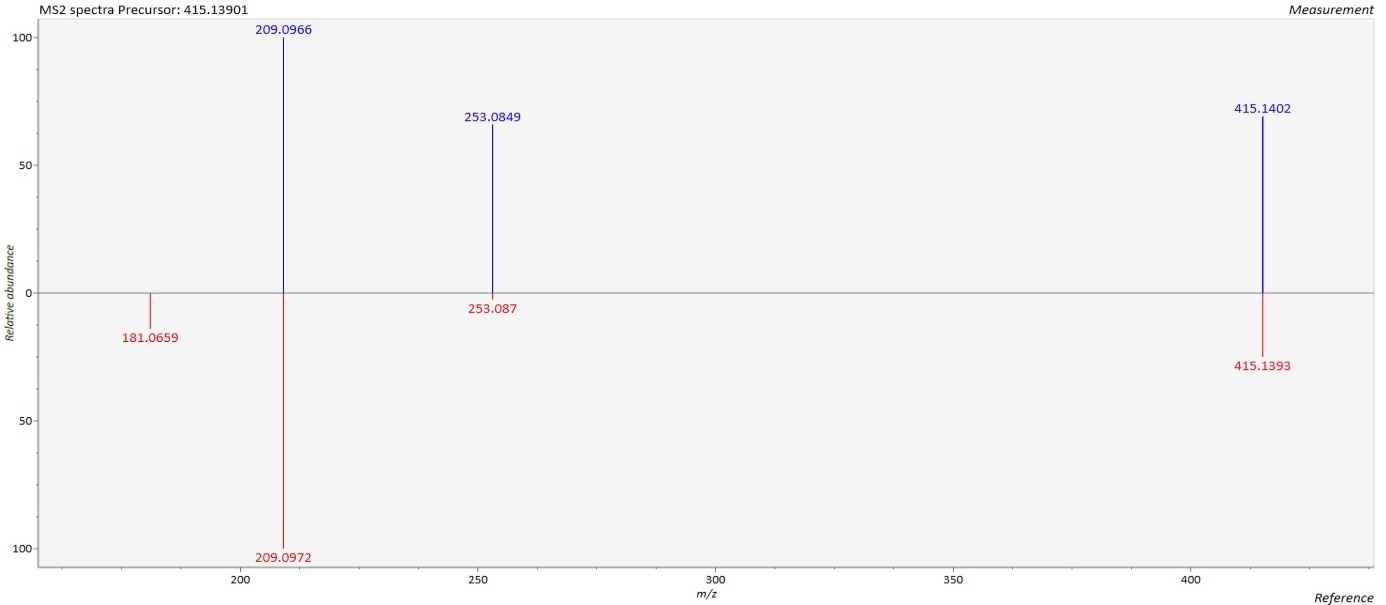


**Fig. S19** MS/MS spectra of Ketoprofen-R63 in ESI-, Library match with in-silico predicted MS/MS spectra


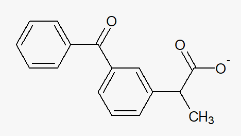

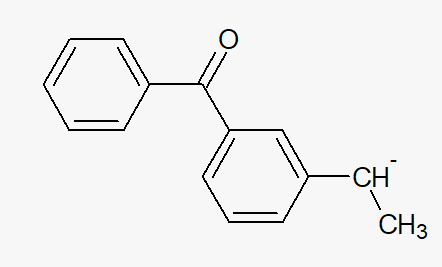

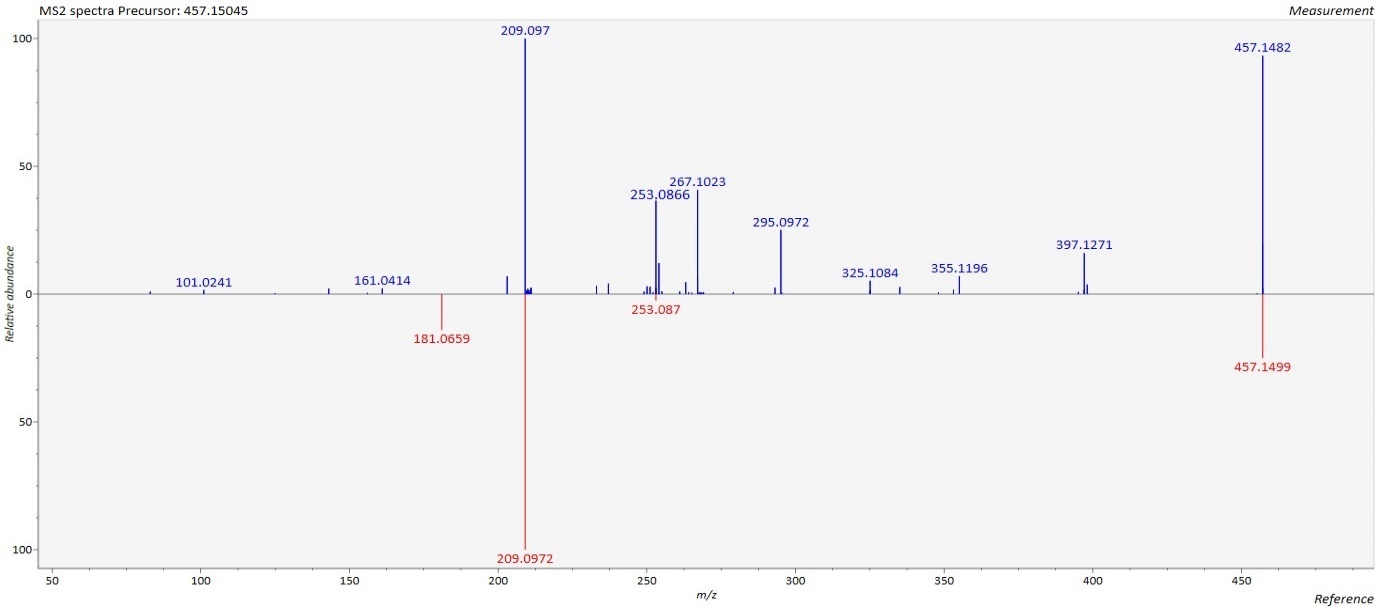


Acetylated

Ketoprofen-Glu

[M-H]^-^

**Fig. S20** MS/MS spectra of Ketoprofen-R81 in ESI-, Library match with in-silico predicted MS/MS spectra


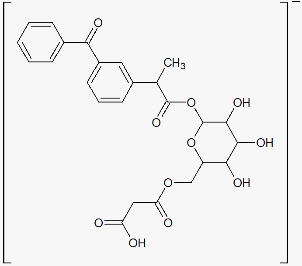

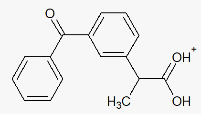


**Fig. S21** MS/MS spectra of Ketoprofen-R87 in ESI+, Library match with in-silico predicted MS/MS spectra

**Fig. S22** MS/MS spectra of Ketoprofen-R93 in ESI-, Library match with in-silico predicted MS/MS spectra

Hydroxylated

Ketoprofen-Glu

[M-H]^-^

**Fig. S23** MS/MS spectra of Ketoprofen-R96 in ESI-, Library match with in-silico predicted MS/MS spectra

Hydroxylated

Ketoprofen-Glu-Glu-Glu-Glu

[M-H]^-^

**Fig. S24** MS/MS spectra of Ketoprofen-R111 in ESI-, Library match with in-silico predicted MS/MS spectra

**Fig. S25** MS/MS spectra of Ketoprofen-LS24 in ESI-, Library match with in-silico predicted MS/MS spectra

**Fig. S26** MS/MS spectra of Ketoprofen-LS25 in ESI-, Library match with in-silico predicted MS/MS spectra

**Fig. S27** MS/MS spectra of Ketoprofen-M648 in ESI-, Library match with in-silico predicted MS/MS spectra

**Fig. S28** MS/MS spectra of Ketoprofen-M835 in ESI+, Library match with in-silico predicted MS/MS spectra

**Fig. S29** MS/MS spectra of Enrofloxacin in ESI+, Library match with in-silico predicted MS/MS spectra

**Fig. S30** MS/MS spectra of Enrofloxacin-R63 in ESI+, Library match with in-silico predicted MS/MS spectra

Methylated

Enrofloxacin

[M+H]^+^

**Fig. S31** MS/MS spectra of Enrofloxacin-R13 in ESI+, Library match with in-silico predicted MS/MS spectra

**Fig. S32** MS/MS spectra of Ciprofloxacin in ESI+, Library match with in-silico predicted MS/MS spectra

**Fig. S33** MS/MS spectra of Enrofloxacin-R2 in ESI+, Library match with in-silico predicted MS/MS spectra

**Fig. S34** MS/MS spectra of Enrofloxacin-M242 in ESI+, Library match with in-silico predicted MS/MS spectra

**Fig. S35** MS/MS spectra of Tetracycline in ESI+, Library match with in-silico predicted MS/MS spectra

**Fig. S36** MS/MS spectra of Tetracycline in ESI-, Library match with in-silico predicted MS/MS spectra

**Fig. S37** MS/MS spectra of Tetracycline-LS2 in ESI+, Library match with in-silico predicted MS/MS spectra

**Fig. S38** MS/MS spectra of Tetracycline-LS3 in ESI+, Library match with in-silico predicted MS/MS spectra

**Fig. S39** MS/MS spectra of Erythromycin in ESI+, Library match with in-silico predicted MS/MS spectra

**Fig. S40** MS/MS spectra of ErythromycinM-1 in ESI+, Library match with in-silico predicted MS/MS spectra

**Fig. S41** MS/MS spectra of ErythromycinM-46 in ESI+, Library match with in-silico predicted MS/MS spectra

**Fig. S42** MS/MS spectra of ErythromycinM-361 in ESI+, Library match with in-silico predicted MS/MS spectra

**Appendix 3.** Normalized Abundance of metabolites

Peaks for parent drugs and metabolites were normalized to 0.1 g of lyophilized samples. Because standards for identified metabolites were not available due to difficulties in their synthesis, only normalized abundances are displayed in Fig. S43-S52. Moreover, some of the parent substances and their metabolites were detected in different ionization modes (ESI+ or ESI-), adding complexity to relative quantification due to conjugation with endogenous molecules. The ionization process efficiency can also be influenced by the structure of newly formed metabolites, either increasing or decreasing because of different retention times, matrix effects, etc. Additionally, even though the extraction method was carefully optimized and validated for parent drugs, it may not be the most suitable for the extraction of formed metabolites due to lower recovery rates, as these metabolites have different physicochemical properties [6]. Therefore, the relative comparison of these abundances, even after normalization, is far from perfect. Nevertheless, from the obtained results, each compound has different uptake kinetics, transformation pathways, and kinetics, depending on the physicochemical properties of the pharmaceutical, sample type, and sampling time [7]. Some of the metabolites are major (e.g., ATE-LS1, SMX-LS1, SMX-R63, KPF-R13, KPF-R81, ENR-R63), whereas others are minor or short-lived (e.g., TC-LS2, TC-LS3, ERY-M1). In particular, in the case of Ketoprofen, the cumulative sum of metabolite abundance is multiple times higher than that of its parent substances. Consistent with our results, a study [8] has reported several metabolites of diclofenac, naproxen, ketoprofen, and mefenamic acid, displaying higher intensities than the parent drugs.

**Fig. S43** Parent drugs and their metabolites in earthworm tissue, S43A) Atenolol, and S43B) Sulfamethoxazole; Percentage value of metabolites relative to the initial peak area of parent drug

**Fig. S44** Atenolol and its metabolite in lettuce roots S44A) Soil environment; S44B) Hydroponic conditions; Percentage value of metabolites relative to the initial peak area of parent drug

**Fig. S45** Ketoprofen and its metabolite in lettuce roots grown in soil environment; Percentage value of metabolites relative to the initial peak area of parent drug

**Fig. S46** Ketoprofen and its metabolite in lettuce roots grown under hydroponic conditions; Percentage value of metabolites relative to the initial peak area of parent drug

**Fig. S47** Sulfamethoxazole and its metabolite in lettuce roots S47A) Soil environment; S47B) Hydroponic conditions; Percentage value of metabolites relative to the initial peak area of parent drug

**Fig. S48** Enrofloxacin and its metabolites in lettuce roots under Hydroponic conditions; Percentage value of metabolites relative to the initial peak area of parent drug

**Fig. S49** Tetracycline and its metabolites in lettuce roots under Hydroponic conditions; Percentage value of metabolites relative to the initial peak area of parent drug

**Fig. S50** Erythromycin and its metabolites in lettuce roots under Hydroponic conditions; Percentage value of metabolites relative to the initial peak area of parent drug

**Fig. S51** Ketoprofen and its metabolites in lettuce leafs under Soil conditions; Percentage value of metabolites relative to the initial peak area of parent drug

**Fig. S52** Sulfamethoxazole and its metabolites in lettuce leafs under Soil conditions; Percentage value of metabolites relative to the initial peak area of parent drug

**References**

1. Mravcová L, Amrichová A, Navrkalová J, Hamplová M, Sedlář M, Gargošová HZ, et al. Optimization and validation of multiresidual extraction methods for pharmaceuticals in Soil, Lettuce, and Earthworms. Environmental Science and Pollution Research [Internet]. 2024; Available from: https://link.springer.com/10.1007/s11356-024-33492-7

2. Song W, Guo M. Residual Veterinary Pharmaceuticals in Animal Manures and Their Environmental Behaviors in Soils. Applied Manure and Nutrient Chemistry for Sustainable Agriculture and Environment. Dordrecht: Springer Netherlands; 2014. p. 23–52.

3. Barron L, Havel J, Purcell M, Szpak M, Kelleher B, Paull B. Predicting sorption of pharmaceuticals and personal care products onto soil and digested sludge using artificial neural networks. Analyst. 2009;134:663.

4. Williams M, Ong PL, Williams DB, Kookana RS. Estimating the sorption of pharmaceuticals based on their pharmacological distribution. Environ Toxicol Chem. 2009;28:2572–9.

5. NEIL S. MATTSON, CARI PETERS. A Recipe for Hydroponic Success. Inside Grower. 2014 Jan;16–9.

6. Martínez-Piernas AB, Nahim-Granados S, Polo-López MI, Fernández-Ibáñez P, Murgolo S, Mascolo G, et al. Identification of transformation products of carbamazepine in lettuce crops irrigated with Ultraviolet-C treated water. Environmental Pollution. 2019;247:1009–19.

7. Madmon M, Zvuluni Y, Mordehay V, Hindi A, Malchi T, Drug E, et al. Pharmacokinetics of the Recalcitrant Drug Lamotrigine: Identification and Distribution of Metabolites in Cucumber Plants. Environ Sci Technol. 2023;57:20228–37.

8. Emhofer L, Himmelsbach M, Buchberger W, Klampfl CW. High-performance liquid chromatography – mass spectrometry analysis of the parent drugs and their metabolites in extracts from cress ( Lepidium sativum ) grown hydroponically in water containing four non-steroidal anti-inflammatory drugs. J Chromatogr A. 2017;1491:137–44.
