# Supplementary material for: Pharmaceutical metabolite identification in lettuce (Lactuca sativa) and earthworms (Eisenia fetida) using liquid chromatography coupled to high-resolution mass spectrometry and in silico spectral library
Source: Anal Bioanal Chem. 2024 Sep 10;416(28):6291–306. doi: 10.1007/s00216-024-05515-2 (PMC11541386; doi:10.1007/s00216-024-05515-2)
Supplement: Supplementary file 3 — Supplementary file3 presents the script for MS2 in silico prediction. Document outlines instructions for running the script, required input data, output details, and information about the generated MS/MS spectral library. (DOCX 23 KB) [file 216_2024_5515_MOESM3_ESM.docx]

**Supplementary Information for**

**Pharmaceutical Metabolite Identification in Lettuce (Lactuca sativa) and Earthworms (Eisenia fetida) Using Liquid Chromatography Coupled to High-Resolution Mass Spectrometry and In Silico Spectral Library**

Jan Fučík^1,*^, Stanislav Fučík^2^, Sascha Rexroth^3^, Marian Sedlář^4^, Helena Zlámalová Gargošová^1^, Ludmila Mravcová^1^

*^1^ Institute of Chemistry and Technology of Environmental Protection, Faculty of Chemistry, Brno University of Technology, Purkyňova 118, 612 00 Brno, Czech Republic*

*^2^* *Faculty of Electrical Engineering and Communication, Brno University of Technology, Technická 3058/10, 616 00 Brno, Czech Republic*

*^3^* *Shimadzu Europa GmbH, Albert-Hahn-Straße 6, 472 69 Duisburg, Germany*

*^4^* *CEITEC Brno University of Technology, Purkyňova 656/123, 612 00 Brno, Czech Republic*

*corresponding author: [*xcfucikj@vutbr.cz*](mailto:xcfucikj@vutbr.cz)

**# chemical_lib**

**## What is chemical_lib**

This project encompasses [1] the code utilized for batch predictions of chemicals using cfm-predict [2]. The repository also includes data specifying the chemicals targeted for prediction and the corresponding results of the predictions.

This repository is dedicated to supporting the article [3], making it a read-only resource.

**## License**

This project is released under the MIT license. For detailed information, please refer to the LICENSE.md file included in the repository.

**## How to run this script?**

**### Prerequisites for run**

- Linux OS

- make

- docker

- python

**- NOTE:** Some Python packages will be automatically installed by pip during the setup process.

**### Necessary data for prediction**

**NOTE:** Repository already contains prepared data, that were used for the article [3].

**File data/chemical_list.csv**

- **Note**: This file encompasses all metabolites, including those with precisely known structures with available SMILES and InChI, as well as metabolites predicted by common metabolic pathways where SMILES and InChI are not available. As the repository already contains data, the file for article [3] serves as an example. This file explicitly specifies for which metabolites the MS^2^ spectral library should be predicted and included in the output library. If a record contains a valid InChI field, the prediction of MS^2^ spectra for the given chemical will be executed. In cases where MS^2^ information is not available, it will be generated as explained in article [3].

- **Format:** CSV with ';' as the separator.

- **File Description:** The data in the file is structured according to the following csv header:

-- Number;Chemical name;Chemical Formula;Monoisotopic mass;[M+H]+;[M-H]-;Smile Code;INCHi;Source;;

--- **Number** - The order of substances during prediction (e.g. 1, 2, 3, etc.)

--- **Chemical name** – The name of the compound or metabolite (e.g. Sulfamethoxazole, Sulfamethoxazole-LS1, etc.)

--- **Chemical Formula** – The chemical formula of the compound (e.g. C_10_H_11_N_3_O_3_S for Sulfamethoxazole)

--- **Monoisotopic mass** - Monoisotopic mass of the compound (e.g. 253.052114 for Sulfamethoxazole)

--- **[M+H]+** - Mass-to-charge ratio of the protonated molecular ion (e.g. 254.059938 as [M+H]^+^ for Sulfamethoxazole)

--- **[M-H]-** - Mass-to-charge ratio of the deprotonated molecular ion (e.g. 252.044289 as [M-H]- for Sulfamethoxazole)

--- **Smile Code** - SMILES (Simplified Molecular Input Line Entry System) is a series of symbols and characters to represent atoms, bonds, and other structural features. The "SMILE Code" you mentioned is likely a SMILES string, which is a specific sequence of characters representing the structure of a molecule. (e.g. CC1=CC(=NO1)NS(=O)(=O)C2=CC=C(C=C2)N for Sulfamethoxazole)

--- **INCHi** - InChI, which stands for International Chemical Identifier, is a character string that represents a chemical substance in a way that is unique to that substance. (e.g. InChI=1S/C10H11N3O3S/c1-7-6-10(12-16-7)13-17(14,15)9-4-2-8(11)3-5-9/h2-6H,11H2,1H3,(H,12,13) for Sulfamethoxazole)

--- **Source** – It indicates the source or origin of the data or information associated with each chemical compound or metabolite. (e.g. Name of prediction Software, Article doi, etc.)

**File data/metabolites_r.csv**

**Note:** This file exclusively contains metabolites predicted by Common Metabolic Pathways, and its purpose is to generate the MS^2^ spectral library for these particular metabolites. This file includes metabolites for which the precise structure is not known. Consequently, only the columns Number, Chemical Name, Monoisotopic Mass, [M+H]+, and [M-H]- are populated, mirroring the format evident in the reference file available in article [3].

- **Format:** CSV with ';' as the separator.

- **File Description:** The data in the file is structured according to the following csv header:

-- Number;Chemical name;Chemical Formula;Monoisotopic mass;[M+H]+;[M-H]-;Smile Code;INCHi;Source;;

--- **Number** - The order of substances during prediction (e.g. 1, 2, 3, etc.)

--- **Chemical name** – The name of the compound or metabolite (e.g. Sulfamethoxazole, Sulfamethoxazole-LS1, etc.)

--- **Chemical Formula** – The chemical formula of the compound (e.g. C_10_H_11_N_3_O_3_S for Sulfamethoxazole)

--- **Monoisotopic mass** - Monoisotopic mass of the compound (e.g. 253.052114 for Sulfamethoxazole)

--- **[M+H]+** - Mass-to-charge ratio of the protonated molecular ion (e.g. 254.059938 as [M+H]^+^ for Sulfamethoxazole)

--- **[M-H]-** - Mass-to-charge ratio of the deprotonated molecular ion (e.g. 252.044289 as [M-H]- for Sulfamethoxazole)

--- **Smile Code** - SMILES (Simplified Molecular Input Line Entry System) is a series of symbols and characters to represent atoms, bonds, and other structural features. The "SMILE Code" you mentioned is likely a SMILES string, which is a specific sequence of characters representing the structure of a molecule. (e.g. CC1=CC(=NO1)NS(=O)(=O)C2=CC=C(C=C2)N for Sulfamethoxazole)

--- **INCHi** - InChI, which stands for International Chemical Identifier, is a character string that represents a chemical substance in a way that is unique to that substance. (e.g. InChI=1S/C10H11N3O3S/c1-7-6-10(12-16-7)13-17(14,15)9-4-2-8(11)3-5-9/h2-6H,11H2,1H3,(H,12,13) for Sulfamethoxazole)

--- **Source** – It indicates the source or origin of the data or information associated with each chemical compound or metabolite. (e.g. Name of prediction Software, Article doi, etc.)

**### Runing prediction**

Install pip dependencies:

make

Run prediction:

make run

**### Output of prediction**

The prediction results are stored in files located in the "data/computed+" and "data/computed-" folders, corresponding to the prediction model utilized (either ESI+ or ESI-).

Given the time-consuming nature of the prediction process, chemicals that already have results in the mentioned folders are skipped to optimize efficiency. If there is a need to recalculate predictions for a specific chemical, it is necessary to delete the corresponding files in the mentioned folders.

**### Final result – MS/MS spectral library**

The final step of the prediction code involves aggregating the data into a library, which is designed for use in a chemical analysis tool [4]. CFM ID [2] employs collision energies (10, 20, and 40 eV) to predict MS2 spectra. However, our script focuses on simulating analyses in a collision energy spread, specifically using 10 and 20 eV. This intentional exclusion of 40 eV is due to the presence of low molecular fragments with lower specificity. The resulting libraries, presented in text format for both ESI+ and ESI- independently, are located in the "out" folder. Notably, for each ionization mode, there exist two distinct libraries. For instance, there is a "library_positive" comprising predictions where SMILES or InChI information was available. Additionally, there is a "metabolite_library_positive," specifically designed for cases where the chemical structure was known, and metabolites were predicted using the Common Metabolic Pathways approach.

**NOTE:** To utilize these libraries in the msp format, it is sufficient to change the file extension from ".txt" to ".msp". We strongly recommend using positive and negative modes separately. In our case, MS-DIAL 4.0 software was employed for data processing and library matching.

**## Links and References**

[1] https://github.com/stenliis/chemical_lib

[2] https://bitbucket.org/wishartlab/cfm-id-code

[3] Identification of pharmaceutical metabolites after the uptake of parent substances by lettuce (Lactuca sativa) and earthworms (Eisenia fetida) by liquid chromatography coupled to high-resolution mass spectrometry using in silico spectral library

[4] Tsugawa, H.; Ikeda, K.; Takahashi, M.; Satoh, A.; Mori, Y.; Uchino, H.; Okahashi, N.; Yamada, Y.; Tada, I.; Bonini, P.; Higashi, Y.; Okazaki, Y.; Zhou, Z.; Zhu, Z.-J.; Koelmel, J.; Cajka, T.; Fiehn, O.; Saito, K.; Arita, M.; Arita, M. A Lipidome Atlas in MS-DIAL 4. Nature Biotechnology, 2020, 38, 1159–1163. https://doi.org/10.1038/s41587-020-0531-2.
